# Safety outcomes and immunological correlates in a prospective clinical trial of immune checkpoint therapy plus debulking surgery for patients with metastatic renal cell carcinoma

**DOI:** 10.21203/rs.3.rs-4331053/v1

**Published:** 2024-11-12

**Authors:** Sangeeta Goswami, Jianjun Gao, Sreyashi Basu, Daniel D. Shapiro, Jose A. Karam, Rebecca Slack Tidwell, Kamran Ahrar, Matthew T. Campbell, Yu Shen, Alexandro E. Trevino, Aaron T. Mayer, Alexsandra B. Espejo, Christian Seua, Marc D. Macaluso, Yulong Chen, Wenbin Liu, Zhong He, Shalini S. Yadav, Ying Wang, Priya Rao, Li Zhao, Jianhua Zhang, Sonali Jindal, Andrew Futreal, Linghua Wang, Nizar M. Tannir, Padmanee Sharma

**Affiliations:** 1 Department of Genitourinary Medical Oncology, The University of Texas MD Anderson Cancer Center, Houston, TX; 2 Department of Urology, The University of Texas MD Anderson Cancer Center, Houston, TX; 3 Department of Translational Molecular Pathology, The University of Texas MD Anderson Cancer Center, Houston, TX; 4 Department of Biostatistics, The University of Texas MD Anderson Cancer Center, Houston, TX; 5 Department of Interventional Radiology, The University of Texas MD Anderson Cancer Center, Houston, TX; 6 Enable Medicine, Menlo Park, California; 7 Immunotherapy Platform, The University of Texas MD Anderson Cancer Center, Houston, TX; 8 Department of Pathology, The University of Texas MD Anderson Cancer Center, Houston, TX; 9 Department of Genomic Medicine, The University of Texas MD Anderson Cancer Center, Houston, TX; 10 Department of Immunology, The University of Texas MD Anderson Cancer Center, Houston, TX; 11 James P. Allison Institute, The University of Texas MD Anderson Cancer Center, Houston, TX

## Abstract

Surgical removal of primary tumors was shown to reverse tumor-mediated immune suppression in pre-clinical models with metastatic disease. However, how cytoreductive surgery in the metastatic setting modulates the immune responses in patients, especially in the context of immune checkpoint therapy (ICT)-containing treatments is not understood. Here, we report the first prospective, non-comparative clinical trial to evaluate the feasibility, clinical benefits, and immunologic changes of combining three different ICT-containing strategies with cytoreductive surgery or biopsy for patients with metastatic clear cell renal cell carcinoma (mccRCC). Based upon baseline evaluation and surgical eligibility after 6 weeks of ICT treatment, 43 patients on this trial proceeded with cytoreductive surgery, while 36 patients who had medical comorbidities preventing surgery or did not have a lesion amenable for surgical resection underwent post-ICT biopsy as specified in the clinical trial protocol, and 25 patients who discontinued study participation due to progressive disease or toxicities or withdrawal of consent did not receive either procedure (total N=104). Our data demonstrated that, in the subgroup of patients receiving the combination of ICT with cytoreductive surgery or biopsy, no additional ICT- or procedure-related toxicities were observed as compared to historical data. The median OS (overall survival) was 54.7 months for patients who received ICT-containing regimens plus cytoreductive surgery (n=43). Immune-monitoring studies with co-detection by indexing (CODEX) identified distinct tumor spatial conformation of cellular subsets as a novel and improved predictor of response to ICT. Importantly, single-cell RNA-sequencing (sc-RNA-seq) data demonstrated that surgical removal of the tumor increased antigen-presenting dendritic cell population with a concurrent reduction in KDM6B-expressing immune-suppressive myeloid cells in the peripheral blood. Together, this study highlighted the feasibility of combining ICT with cytoreductive surgery in a metastatic setting and demonstrated the potential enhancement of immune responses following ICT plus cytoreductive surgery in patients with metastatic disease.

## Introduction

Patients diagnosed with stage IV solid tumors, characterized by multiple metastatic lesions throughout the body, have traditionally been considered ineligible candidates for surgical interventions targeting the primary tumor or any metastatic sites^1, 2^. Nonetheless, in pre-clinical murine models bearing metastatic disease, removal of the primary tumor resulted in the reversal of tumor-mediated immune suppression^3^, highlighting the possibility of improved response to immune-based therapy in the remaining metastatic disease. Additionally, a retrospective study of patients with metastatic melanoma treated with immune checkpoint therapy (ICT) demonstrated that patients who subsequently underwent complete surgical resection of metastases had improved survival compared to those with incomplete resection, suggesting that surgery may provide a survival benefit in the setting of ICT^4^. However, the concept of debulking surgery to remove a single lesion (the primary tumor-bearing organ or a metastatic lesion) to enhance anti-tumor response with continued post-surgery ICT to treat other metastatic sites of disease in the same patient has not previously been investigated in a prospective study.

To investigate the safety as well as potential clinical and biological outcomes of ICT plus cytoreductive surgery in the metastatic disease setting, we designed an open-label, pilot, non-comparative clinical trial (NCT02210117) with three different ICT-containing regimens plus cytoreductive surgery, or biopsy if a patient is not eligible for surgery, in patients with mccRCC (N=104). Each patient on this trial was given 6 weeks of systemic therapy consisting of nivolumab, nivolumab plus bevacizumab, or nivolumab plus ipilimumab, before surgery or biopsy, followed by nivolumab maintenance therapy for up to 2 years until disease progression, toxicity, or withdrawal from the protocol. The primary endpoint of the study was safety for all patients, and the secondary endpoints included best overall response, progression-free survival (PFS), overall survival (OS), and correlative immunological responses. We noted that a combination of ICT plus cytoreductive surgery or biopsy in the metastatic setting is safe. The median OS was 54.7 months for patients who received ICT plus surgery (n=43). Additionally, an *ad hoc* non-comparative analysis showed a median OS of 23.5 months for patients who received ICT without surgery (n=61).

Baseline and post-treatment tissue samples and peripheral blood samples were analyzed to assess immune-genomic markers correlating with clinical benefit. Tumor interferon-gamma (IFN-γ) gene signature and tertiary lymphoid structure (TLS) gene signature correlated with improved clinical response. However, we observed a cohort of patients with high IFN-γ and TLS gene signatures who did not respond to ICT. Analyses of spatial conformation of tumor immune cell subsets in patients with high IFN-γ and high TLS gene signatures including those without clinical response to ICT delineated distinct cellular distribution and enrichment of certain cellular neighborhoods that accurately correlated with clinical response to ICT. Importantly, longitudinal analyses of matched peripheral blood samples at a single-cell level by sc-RNA-seq demonstrated increased conventional dendritic cell population with a concurrent reduction in KDM6B expressing immune-suppressive myeloid cells in patients who underwent surgical removal of the tumor compared to patients who did not.

Overall, we report herein the safety and immune correlative data from this prospective pilot trial. Our data demonstrated the feasibility of combining ICT with cytoreductive surgery in mccRCC, highlighted the importance of the spatial distribution of immune cell subsets in determining response to ICT, and demonstrated the effect of cytoreductive surgery in modulation of the immune responses in the metastatic disease setting.

## Results

### Combination of ICT plus surgery or biopsy is safe and feasible in the metastatic setting

**Fig. 1.**
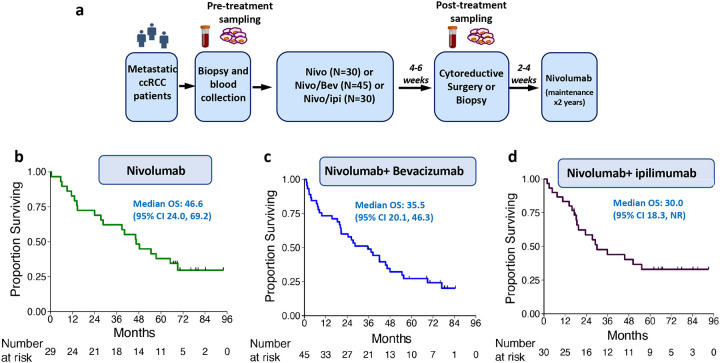
Combination of ICT plus surgery is safe and feasible in the metastatic setting **(a)** Schema for clinical trial NCT02210117. Patients with mccRCC underwent baseline tumor biopsy and blood sample collection before being randomly assigned to receive nivolumab (n=30), nivolumab + bevacizumab (n=45), or nivolumab + ipilimumab (n=30) for a total of 6 weeks. Four to six weeks after the ICT treatment, based upon evaluation by medical oncologists and urologists specialized on RCC, patients underwent either cytoreductive surgery or tumor biopsy. Two to four weeks after cytoreductive surgery or biopsy, nivolumab was given as maintenance therapy to each patient for up to 2 years or until disease progression or intolerable toxicities or withdrawal from the protocol. Tissue and blood samples were collected at pre-ICT treatment and at the time of surgery or biopsy (4–6 weeks after the initial 6 weeks of ICT treatment) for correlative studies. **(b)** Overall survival (OS) in Arm A (nivolumab). **(c)** OS in Arm B (nivolumab + bevacizumab). **(d)** OS in Arm C (nivolumab + ipilimumab).

Between July 2015 and March 2018, 105 patients were randomly assigned to receive nivolumab (n=30), nivolumab+ bevacizumab (n=45), or nivolumab + ipilimumab (n=30) for a total of 6 weeks, then underwent cytoreductive surgery or tumor biopsy, followed by maintenance nivolumab therapy for up to 2 years. Baseline and post-treatment tissue samples and peripheral blood samples were collected for immunologic and genomic analyses (Fig. 1a, Fig. S1a,b). One patient was not treated in the nivolumab arm due to inadvertent randomization prior to completing screening and ultimately was not eligible for enrollment in the trial (and thus n=104 were used for clinical outcome analysis). After enrollment into the trial, patients were concurrently evaluated by medical oncologists and urologists specialized in RCC, at baseline and after completing 6 weeks of systemic therapy, to assess the suitability of debulking surgery or biopsy. Based upon baseline evaluation and surgical eligibility after 6 weeks of ICT treatment, 43 patients on this trial proceeded with cytoreductive surgery, while 36 patients who had medical comorbidities preventing surgery or did not have a lesion amenable for surgical resection underwent post-ICT biopsy as specified in the clinical trial protocol, and 25 patients who discontinued study participation due to progressive disease (n=17) or toxicities (n=3) or withdrawal of consent (n=5) did not receive either procedure (Fig. S1a, S1b). All (N=104) patients were evaluated for safety and clinical responses with a median follow-up of 76.1 months. Patient characteristics including age, gender, performance status, and international metastatic RCC database consortium (IMDC) prognostic risk group were described in Table S1. Overall, the ICT-related toxicity profile for this study (Table 1 and Table S2) was expected and comparable to previously published data on ICT monotherapy or combination therapy in mccRCC^5, 6^. For the patients who underwent cytoreductive surgery (n=43), treatment with ICT did not result in any delays in surgery or wound complications. Specifically, the 90-day surgical complications among patients who received combination treatment with ICT plus surgery in this trial was 14% (6/43) as compared to historical data for cytoreductive surgical complications which have ranged between 12–57%^7^ (Table 2 and Table S3).

Clinical responses per RECISTv1.1 criteria were assessed at 12 weeks after treatment initiation as best overall response. We assessed BOR in patients who still had metastatic lesions that could be followed by imaging studies. The BOR at 12 weeks (excluding the surgery effect) was 34% for all patients, 45% in the nivolumab arm, 36% in the nivolumab + bevacizumab arm, and 30% in nivolumab + ipilumimab arm (Table S4). Additionally, we performed *ad hoc* non-comparative analyses examining outcomes for patients separately for those who received surgery and those who did not. Patients with ICT plus surgery had BOR of 79% in all ICT arms combined (Table S4). For individual ICT arms, the BOR in the group of patients who were treated with ICT plus surgery was 86% in the nivolumab arm, 81% in the nivolumab + bevacizumab arm, and 69% in the nivolumab + ipilimumab arm (Table S4). These unusually high response rates are partly because cytoreductive surgery removed target lesions in some patients. After adjusting for the effect of surgical resection of targeted lesions, the BOR was 57% in the nivolumab arm, 56% in the nivolumab + bevacizumab arm, 38% in the nivolumab + ipilimumab arm, and 51% in all arms combined in patients who received ICT plus surgery (Table S4). For patients who received ICT without surgery (n=61), the BOR was 33% in the nivolumab arm, 24% in the nivolumab + bevacizumab arm, 24% in the nivolumab + ipilimumab arm, and 26% in all arms combined (Table S4). Since this study is a non-comparative clinical trial with primary endpoint of safety, no statistical comparisons were made between different treatment arms of ICT or between the surgery group and non-surgery group.

With a median follow-up time of 76.1 months, the median OS was 46.6 months (95% confidence interval [CI] 24.0, 69.2) in the nivolumab arm, 35.5 months (95% CI 20.1, 46.3) in the nivolumab + bevacizumab arm, and 30 months (95% CI 18.3, not reached [NR]) in the nivolumab + ipilimumab arm (Fig. 1b–d). The median PFS was 12.4 months (95% CI 5.5, 16.8) in the nivolumab arm, 7.6 months (95% CI 4.8, 9.1) in the nivolumab + bevacizumab arm, and 8.6 months (95% CI 2.1, 16.8) in the nivolumab + ipilimumab arm (Fig. S2 a–c). Continuing the *ad hoc* non-comparative analyses of patients treated with ICT plus surgery, the median OS was 54.7 months (95% CI 40.2, NR) (Fig. S3a); and the median PFS was 12.4 months (95% CI 8.0, 18.3) (Fig. S3b). For all patients who received ICT without surgery (n=61), the median OS was 23.5 months (95% CI 14.3, 35.5) (Fig. S3c) and the median PFS was 4.7 months (95% CI 2.0, 7.8) (Fig. S3d). Representative images of the clinical response to ICT plus surgery are shown in Fig. S3e.

Together, these data demonstrated the safety and feasibility of combining ICT with cytoreductive surgery or biopsy in mccRCC. Interestingly, we noted that patients who could undergo surgery appeared to have durable clinical outcomes, although this is based upon an *ad hoc* evaluation instead of pre-conceived analysis of randomized data and thus may be, at least in part, a reflection of selection of “fit” patients for surgery.

### Patients with *PBRM-1* and *SETD-2* mutations demonstrate improved clinical response

An important secondary objective for this pilot trial was to perform immune monitoring analyses to assess genomic and immunologic changes that correlate with clinical response, which was defined as partial response (PR), stable disease (SD) or progressive disease (PD). Among a total of 104 patients on this trial, we were able to obtain tissue samples from 94 patients for immune monitoring studies. The treatment allocation and the number of patients who had tissue available for analysis are shown in the CONSORT diagram in Fig. S4a.

**Fig. 2.**
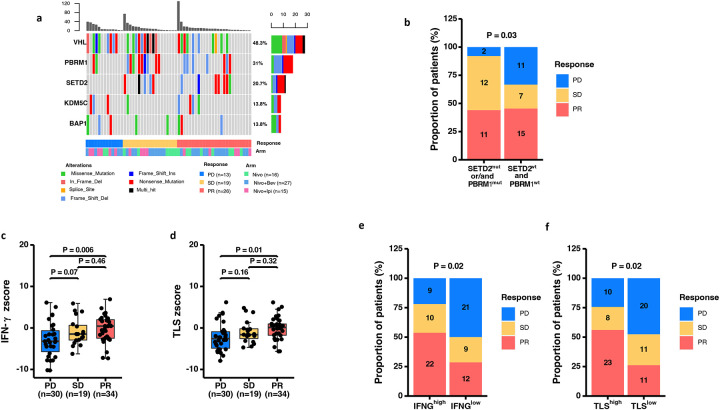
Identification of immuno-genomic biomarkers from tumor tissues of mccRCC patients. **(a)** Oncoplot showing the somatic mutation landscape of the top 5 mutated genes [from TCGA Kidney Renal Clear Cell Carcinoma (KIRC)]. A total of 58 tumor tissue samples were analyzed and each column represents a patient. The color bar at the bottom shows response for each patient (PD=progressive disease, SD=stable disease, PR=partial response). The genes are listed on the left and their respective frequencies are listed on the right of the heatmap. The colored rectangles indicate different types of somatic mutations and the key identifying each mutation type is shown at the bottom of the heatmap. The bar plot on the top shows the somatic mutation count for each patient. The bar plot on the right side shows the counts of mutations for each gene and the colors in the bar plots correspond to the colors showing mutation types in the body of the heatmap. **(b)** Stacked bar plot showing a positive association of genomic signature (mutations in *PBRM1* or *SETD2* genes) with clinical responses. Patients (n=58) were stratified into Mut (patients with mutations in *SETD2* or *PBRM1,* n=25) and WT (patients with wild-type *SETD2* and *PBRM1* genes, n=33) groups (p= 0.03). **(c-d)** Box plots showing association of IFN-γ signature **(c)** and TLS signature **(d)** with clinical responses (n=83). Box plots represent the median, interquartile range and the whiskers represent 1.5 x the upper and lower interquartile range values. Welch’s ANOVA test across the 3 groups for IFN-γ signature (p= 0.025) and TLS signature (p= 0.039). Stacked bar plot showing a positive association of IFN-γ signature (e) and TLS signature (f) with response. Patients (83) were stratified into IFNG^high^ (n=41) and IFNG^low^ (n=42) groups (p= 0.02) **(e)** or into TLS^high^ (n=41) and TLS^low^ (n=42) **(f).** Statistical significance was calculated using Welch’s ANOVA,Welch’s t-test and Fisher’s exact test for comparing z scores in 3 or more unpaired groups, comparing z scores in 2 unpaired groups and comparing group counts, respectively. p < 0.05 was considered statistically significant. The following color scheme is used in all figures showing biological response groups; PD=blue, SD=yellow, and PR=red.

Whole exome sequencing (WES) of available tumor samples (n=58) with paired peripheral blood mononuclear cells (PBMC), was performed to identify tumor-specific mutations correlating with clinical benefit. WES demonstrated VHL, PBRM1, and SETD2 to be the three most commonly mutated genes (Fig. 2a and Fig. S4b). Most of the *PBRM1* mutations were frameshift deletions and nonsense mutations, with few missense mutations whereas the majority of *SETD2* mutations were nonsense mutations (Fig. 2a). Importantly, mutations in either *PBRM1* or *SETD2* were enriched among patients with PR or SD (Fig. 2b). Analysis of copy number alterations (CNA) of top 20 most commonly mutated genes in ccRCC did not show any correlation of CNA with clinical response (Fig. S4c). Next, we calculated tumor mutational burden (TMB) based on counts of somatic mutations per megabase (Mb) of the captured region. We did not note any correlation of TMB with clinical response (Fig.S4d). Additionally, we did not detect any correlation of predicted neoantigen load with clinical benefit (Fig. S4e).

IFN-γ gene signature is often correlated with clinical response in various tumor types, thus multiple studies are currently investigating the use of IFN-γ gene signature as a predictive biomarker^8, 9, 10^. Therefore, we performed NanoString gene expression analysis to evaluate IFN-γ gene signature in available pre-treatment (n=83) samples. Pre-treatment samples were pooled from all patients since the tumor tissues were not exposed to ICTs. Analysis of pre-treatment samples demonstrated a correlation of IFN-γ gene signature with clinical responses in patients receiving ICT with nivolumab as a common backbone across the three treatment groups (Fig. 2c). Similarly, patients with PR had higher TLS-gene expression scores as compared to patients with PD (Fig. 2d). Next, sequential analyses of gene expression in matched pre- and post-treatment samples (n=60) also showed higher IFN-γ gene signature and TLS-gene signature in the post-treatment samples (Fig. S5a–b) and differential gene expression (DEG) analysis showed upregulation of genes including CD8A, Granzyme K (GZMK), CXCL13, CCL19, CCR7, and PDCD1 (Fig. S5c) suggest a pro-inflammatory change in the tumor immune microenvironment following ICT-based therapy. Although IFN-γ and TLS gene signatures correlated with improved response, we observed a cohort of patients with high IFN- γ and TLS gene signatures who did not respond to ICT (Fig. 2e–f).

### Spatial distribution of immune cell subsets is a critical determinant of response to ICT

**Fig. 3.**
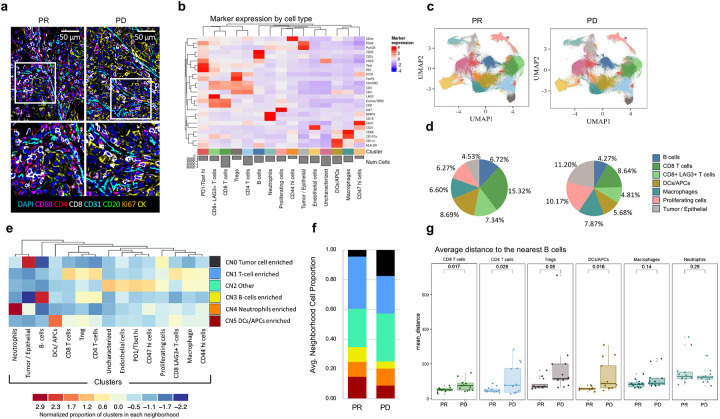
Spatial distribution of immune cell subsets using CODEX. (a) Multiplex IF of immune cell aggregate for a representative partial response (PR) and progressive disease (PD) case showing staining for CD4, CD8, CD20, CD68, Ki-67, Pan CK and CD31 (PR, n=4; PD, n=4) (b) Heatmap of the average expression of 25 markers in the different cell clusters is shown. (c) The UMAP plots for PR and PD cases shows a total of 15 cell clusters that were validated by manual inspection of multiplexed immunostains from a 25 markers panel staining using CODEX. Color- code correspond to those for each cluster in the fig. (b). (d) Pie graph shows percentages of most abundant cell clusters in PR and PD cases. Neighborhood (CN) are defined based on the presence of the 15 validated clusters. A total of six CN are identified. The stacked bar graph shows distribution of each cell neighborhood between PR (n=4) and PD (n=4) cases. (g) The bar plots shows average cell distance to nearest B cells from different cell subsets, each dots represent each region of interest from all PR and PD cases.

We hypothesized that the lack of correlation between IFN-γ and TLS gene signatures and clinical responses in a subset of patients may be due to the fact that gene expression data do not provide information on cellular spatial distribution; therefore, we performed co-detection by indexing (CODEX) analyses to evaluate cellular phenotype and distribution pattern in patients who had high IFN-γ and TLS gene signature associated with partial clinical response (PR, n=4) as compared to patients who had high IFN-γ and TLS gene signature associated with progressive disease (PD, n=4). CODEX, a multiplexed cytometric imaging approach^11, 12^ allows spatial analyses of single cells and their distribution within the cellular neighborhood. We assessed the cell subsets assigned by Leiden-based clustering on a staining panel consisting of 25 markers and the annotated clusters were further validated by manual inspection of multiplexed immunostains on images. This led to the identification of 15 unique cell clusters comprising of CD8 and CD4 T cells, neutrophils, tumor/epithelial cells, blood vessels, DC/APCs, macrophages, and other immune clusters. (Fig. 3a–c). We noted a distinct pattern of cell subsets in patients with high IFN-γ and TLS gene signatures who responded to ICT compared to patients who had high IFN-γ and TLS gene signatures but did not respond to ICT. While the percentage for B cells, CD8 T cells, and dendritic cells (DCs) was higher in PR cases, more tumor/epithelial cells were noted in the PD cases (Fig. 3d). To understand the spatial organization of the cell clusters, we assessed cell neighborhoods based on the 15 annotated cell clusters and identified 6 distinct cellular neighborhoods (CNs) (Fig. 3e). Enrichment of CD8 T cell neighborhood (CN1), B cell neighborhood (CN3) and DCs/APC cell neighborhood (CN5) were identified in PR while tumor cell neighborhood (CN0) was more dominant in PD (Fig. 3f). To investigate cell-cell interactions, we conducted a preliminary analysis using a single pair of PR and PD cases. This analysis identified and validated 13 clusters (Fig. S6a). Among these cell types, we observed stronger intercellular interactions between CD4, CD8 T cells, B cells, and DCs in the PR case compared to the PD case within the 3–100-micron range^13^ (Fig. S6b). Next, we expanded our initial cell-cell interaction analysis to include 25 regions of interest across all 8 patient samples (PD=4, PR=4), and a spatial cellular graph was constructed for each cell to its 10 closest neighbors. The volcano plots showed interactions between tumor cells and CD4 T cells as well as DCs with B cells in PR while interactions between CD8 and Tregs as well as B cells and macrophages were seen in PD (Fig. S6c). Additionally, we computed the average minimal cell distance between different pairs of cellular subsets in PR and PD cases. Our analysis showed that B cells were closely aggregated with CD8, CD4, and DCs in PR compared to PD cases (Fig. 3g, Fig. S7). Together, this data demonstrated that although IFN-γ response is required to mount an immune response, the spatial organization and distribution of immune cell subsets finally dictate the outcome to ICT.

### Cytoreductive surgery is associated with the pro-inflammatory skewing of the peripheral immune status in patients with mccRCC

**Fig. 4.**
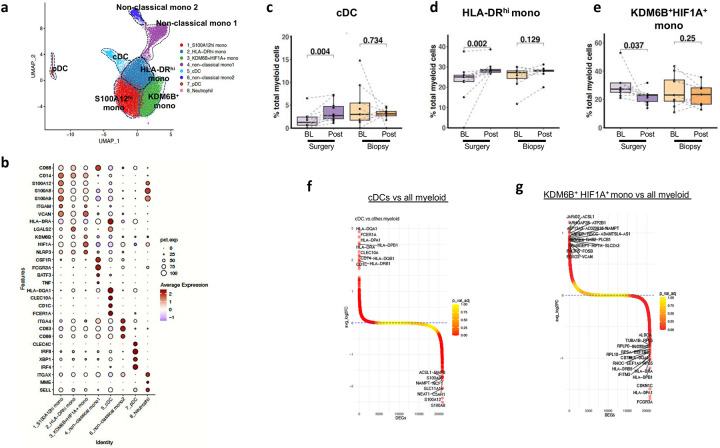
Pro-inflammatory changes in innate immune status after cytoreductive surgery. (a) UMAP projection of myeloid cell subclusters in peripheral blood. (b) Dot plot indicating the average expression of indicated genes as well as the percentage of cells expressing the gene to define myeloid subclusters. (c-e) Box plots of frequency of cDCs (c), HLA-DR^hi^ monocytes (d), and KDM6B^+^HIF1A^+^ monocytes (e), from patients who underwent surgery (n = 10) or biopsy (n= 9) comparing post-surgery or post-biopsy samples to baseline (BL). P values indicated were analyzed using Wilcoxon signed-rank test. (f-g) Identity of differentially expressed genes (DEGs) in cDCs (f) and KDM6B^+^HIF1A^+^ monocytes (g) from PBMCs.

Based on our *adhoc* analyses, we noted that patients with mccRCC who underwent surgery had a median OS of 54.7 months. Further, metastatic models in preclinical settings previously showed that removal of the primary tumor reverts tumor-mediated immune suppression^3^. Therefore, to garner insight into how cytoreductive surgery in mccRCC might modulate the immune system and enhance response to ICT, we performed sc-RNA-seq analyses on matched peripheral blood samples from mccRCC patients (n=38). We assessed the changes in the immune cell subsets in patients who underwent surgery (n=20, baseline=10, post-surgery=10) and compared them to patients who underwent biopsy (n=18, baseline=9, post-biopsy=9). Using graph-based clustering of uniform manifold approximation and projection (UMAP), we identified 4 major immune cell clusters (T cell, NK cell, myeloid cell, and B cell) based on the expression of canonical genes (Fig. S8a–b), Cluster frequency of the major immune cell subsets showed no significant difference between post-surgery or post-biopsy group when compared to their corresponding baseline (Fig. S8c). To identify changes in T or NK cell subsets, we further sub-clustered them into 15 clusters (Fig. S9a–b) and myeloid cell subsets into 8 clusters (Fig. 4a–b). Although there were no significant differences in T or NK cell subsets (Fig. S9c), we identified significant changes in the frequency of immune-stimulatory and immune-inhibitory myeloid cell subsets in the peripheral blood of patients who underwent cytoreductive surgery (Fig. 4c–e & S10). We noted the presence of S100A12^hi^ monocytes (C1), HLA-DR^hi^ monocytes (C2), KDM6B^+^HIF1A^+^ monocytes (C3), non-classical monocytes (C4 and C6), conventional dendritic cells (cDCs) (C5), plasmacytoid DCs (pDCs) (C7) and neutrophils (C8) (Fig. 4a–b). Further evaluation of the myeloid cell subsets revealed that the abundance of the antigen-presenting cells including cDCs (C5) and HLA-DR^hi^ monocytes (C2) increased significantly in post-surgery samples but not in post-biopsy samples compared to their matched baseline (Fig. 4c–d). Importantly, we noted a concurrent decrease in a KDM6B^+^HIF1A^+^ immune-suppressive myeloid cell subset following cytoreductive surgery but not in patients who underwent biopsy compared to the matched baseline samples (Fig. 4e). cDCs are known antigen-presenting cells, which are critical in mounting T cell-mediated immune response. Differential gene expression (DEG) analyses further showed higher expression of HLA molecules in the cDC subsets (C5), confirming their antigen-presenting capacity (Fig. 4f). We recently characterized the immune-suppressive nature of KDM6B expressing myeloid cells which inhibit T cell-mediated antitumor immunity^14^. DEG analyses also demonstrated higher expression of other immune-suppressive genes such as HIF1A in this subset (Fig. 4g, Table S9). Cumulatively, this data suggests that ICT treatment followed by cytoreductive surgery is associated with a pro-inflammatory skewing of the peripheral immune signature with increasing cDC population and HLA-DR^hi^ monocytes and a concurrent reduction in KDM6B expressing immune-suppressive myeloid cells in the peripheral blood. Thus, highlighting the potential reversion of tumor-mediated immune-suppression in patients with mccRCC following cytoreductive surgery which is possibly linked to 2-year overall survival of 84% of cases seen in this cohort of patients.

## Discussion

Surgery for patients with mccRCC has been explored as a treatment approach through surgical removal of the primary tumor-bearing kidney (nephrectomy) and/or metastasis (metastasectomy), which has been referred to as cytoreductive or “debulking” surgery. The combination of systemic therapy, such as cytokine therapy or targeted therapy, with cytoreductive surgery in mccRCC patients remains controversial due to the limited and/or conflicting evidence demonstrating a therapeutic benefit. ICT^15, 16, 17, 18, 19^ enhances anti-tumor T cell responses and provides durable clinical benefits in patients with mccRCC^5, 6^. However, the value of combining ICT with debulking surgery in mccRCC is not known and currently, there is no data to prospectively evaluate the feasibility and benefits of ICT combined with surgery for patients with metastatic disease. Therefore, we designed the pilot clinical trial to test the safety and feasibility, as well as the clinical and biological outcomes of ICT with cytoreductive surgery or biopsy for patients with mccRCC.

The treatment landscape of mccRCC has changed dramatically since the initiation of this trial. While ICT agents such as nivolumab and ipilimumab were not FDA approved before initiation of this trial, bevacizumab was used in clinical practice. This trial enrolled the first patient in July 2015 before nivolumab and ipilimumab were approved by the FDA for metastatic RCC. Therefore, this trial offered patients with mccRCC the promising clinical benefits of nivolumab, bevacizumab, and ipilimumab. Although nivolumab plus ipilimumab is now FDA approved as front line and nivolumab monotherapy is approved for subsequent lines of therapy, none of this has been tested in a pre-surgical setting in combination with cytoreductive surgery in mccRCC. Therefore, this trial allowed to test the safety and feasibility of combining cytoreductive surgery (or biopsy) plus ICT with nivolumab as a common backbone across the three treatment groups. This non-comparative trial was designed to describe rather than compare clinical efficacy between different treatment arms of ICT. However, it is worth noting that in our trial the nivolumab arm has higher RR and PFS as compared to the nivolumab plus bevacizumab arm and the nivolumab plus ipilimumab arm likely due to due to the nivolumab arm has 1) younger patients; 2) more untreated patients; 3) fewer metastatic sites; and 4) less bone metastasis and more lung metastasis (Table S1). Further, it is important to highlight that clinical response was assessed by 12 weeks in our trial, whereas other studies e.g. IMMotion 151^20^ and JAVELIN Renal 101^21^ assessed clinical response at maximal response time, which could explain the difference in the CR rate between our trial and other studies.

Our data demonstrated that ICT can be safely combined with cytoreductive surgery for the treatment of patients with mccRCC who have multiple metastatic lesions. In addition, this trial was not statistically designed to compare clinical outcomes for patients who received ICT plus surgery versus patients who received ICT without surgery. The *ad hoc* non-comparative analyses examining outcomes for patients separately for those who received surgery and those who did not receive surgery. We noted that a median OS of 54.7 months for patients who received ICT plus surgery. For those patients who received ICT only without surgery, the median OS was 23.5 months. This is likely due to the fact that the ICT plus surgery group enriched clinically “fit” patients who were candidates for surgery. Similarly, we noted a generally noted a high BOR rates (38–57%, Table S4) for patients who received ICT plus surgery. For those patients who received ICT only without surgery, the BOR rates were 24–33% (Table S4). This likely due to the fact the ICT without surgery group had a higher proportion of patients with >3 metastatic sites and with previous localized and systemic therapies (Table S5).

Prior to the ICT era, cytoreductive surgery has been combined with either cytokine therapy^5, 6, 8^ or targeted therapy^7, 22^ in clinical trials for patients with metastatic ccRCC, the reported median OS in these studies was generally less than 17 months (Table S6). The prolonged survival in patients treated with ICT plus surgery in our study could be due to multiple factors including ICT as a superior therapy compared to cytokine therapy and targeted therapy, improved synergy between ICT and cytoreductive surgery, and selection bias for patients who were “fit” for surgery. Therefore, future prospective, randomized, controlled trials will need to test the hypothesis of whether cytoreductive surgery adds efficacy with ICT.

Over the past few years, much effort has been undertaken to identify biomarkers to predict clinical response to ICT in mccRCC^22, 23, 24, 25, 26, 27, 28^. In our study, we identified both tumor intrinsic (*PBRM1/SETD2* mutations) and extrinsic (IFN-γ and TLS gene signature) components correlating with response to ICT in mccRCC. Importantly, we demonstrated that although IFN-γ response is required to drive an immune response, the spatial distribution of immune cell subsets finally regulate the outcome to ICT. Thus, this study provided a novel insight demonstrating the spatial organization of immune cell subsets within the tumor microenvironment as a critical factor dictating response to ICT. Factors regulating differential spatial conformation of cellular neighborhoods within the tumor immune microenvironment will require further investigations.

The clinical data, coupled with increased peripheral antigen-presenting cells and reduction in KDM6B expressing immune-suppressive myeloid cell subsets in patients who underwent surgical removal of the tumor provided the potential mechanistic insight into immune modulation following cytoreductive surgery in a metastatic setting. This data led to the hypothesis that antigen release during cytoreductive surgery could potentially enhance anti-tumor immunity in patients, which will need to be further interrogated.

Overall, our clinical and translational data may serve as a foundation to guide larger randomized, comparative clinical trials for further investigation of ICT plus cytoreductive surgery for patients with mccRCC and other tumor types as a combinatorial strategy to enhance response to ICT.

## MATERIALS AND METHODS

### Study population and trial design.

The patients in this study included adults with histologically confirmed metastatic clear cell RCC with measurable disease who were eligible for cytoreductive nephrectomy, metastasectomy or post-treatment biopsy. In addition, patients needed to have good performance status and adequate organ functions. Patients with organ allografts, serious autoimmune diseases, active human immunodeficiency virus (HIV), acquired immunodeficiency syndrome (AIDS), hepatitis B virus (HBV), hepatitis C virus (HCV), uncontrolled hypertension, or grade 2 or higher proteinuria were excluded from this study. In addition, patients who were previously treated with anti-CTLA-4, anti-PD1, or bevacizumab were excluded from this trial. Furthermore, patients on systemic immune suppression medications such as high-dose steroids (e.g.,>10 mg prednisone daily or equivalent) or infliximab were also excluded from this study. This trial was a pilot, non-comparative, randomized study (NCT02210117) with a combination of three different ICT-containing strategies with cytoreductive cytoreductive surgery or biopsy for the treatment of patients with mccRCC (Fig. 1a). The trial was approved by the institutional review board (IRB) at the University of Texas MD Anderson Cancer Center. Written informed consent was obtained from all patients for participation in the trial. In addition, all patients provided informed consent for the IRB-approved laboratory protocol MDACC PA13–0291, and all blood and tumor samples used for correlative studies were collected under this protocol. Patients on this trial underwent baseline tumor biopsy and blood sample collection for research use.

Patients were randomized in a 2:3:2 fashion onto this trial to receive nivolumab (n=30) [intravascular (IV) 3 mg/kg every 2 weeks x3 doses], or nivolumab + bevacizumab (n=45) (IV 10 mg/kg every 2 weeks x3 doses), or nivolumab + ipilimumab (n=30) (IV 1 mg/kg every 3 weeks x2 doses) for a total of 6 weeks. Of note, when this trial was designed, there were other studies with nivolumab, or nivolumab plus ipilimumab, but no combination of nivolumab plus bevacizumab at that time. We decided to allocate more patients in the nivolumab plus bevacizumab arm to gather more clinical and biological data on this combination (nivolumab plus bevacizumab) based upon the decision from our group and the sponsoring company (for clinical convenience and financial practicality). Patients were concurrently evaluated by medical oncologists and urologists specialized on RCC, at baseline and after completing 6 weeks of ICT therapy, to assess the suitability of debulking surgery or biopsy. For this purpose, imaging studies such as CT scans were performed at 12 weeks (after 6 weeks of ICT treatment) and read by a collaborating radiologist to assess clinical responses and assist the decision for cytoreductive surgery or biopsy for each patient. Two to four weeks post cytoreductive surgery or biopsy, nivolumab maintenance therapy was given to each patient for up to 2 years or until disease progression or intolerable toxicities or withdrawal from the protocol (Fig. 1a).

Of note, cytoreductive surgery was decided based upon the following criteria (in addition to consent from the patient): 1) Patients should have a resectable primary tumor; 2) ECOG performance score of 0 or 1; 3) Low surgical risk, i.e. absence of significant co-morbid illnesses; 4) Absence of uncontrolled CNS or uncontrolled spine metastasis; 5) Absence of multiple liver metastasis; 6) Absence of multiple bone metastasis; 7) Patients should be candidates for planned systemic therapy; 8) Patients should not have dominant sarcomatoid, transitional cell, or collecting duct carcinoma histology; 9) No active infection, i.e. negative culture for any previously active infection; 10) Patients should have a predicted adequate renal function after nephrectomy; 11) Patients should not have more than 2 organs involved with metastases.

Ninety-day surgical complications were defined according to the Clavien-Dindo classification system^29^. Complications were considered intraoperative if they occurred between the time from the induction of anesthesia to the time when the patient left the post-anesthesia recovery unit. Surgical approach was not mandated by the trial design and was at the discretion of the primary surgeon.

The clinical outcomes of patients were reported but the trial was not designed or powered for comparison between the treatment arms. The primary endpoint of this trial was safety and the secondary endpoints include best overall response, progression free survival (PFS), and overall survival (OS), and correlative immunologic changes. Clinical responses were definedper RECISTv1.1 criteria and assessed at 12 weeks as complete response (CR), partial response (PR), stable disease (SD), or progression of disease (PD). BOR was defined as response status at 12 weeks. These responses were confirmed by another restaging studies about 3 months later (except in patients with apparent rapid disease progression or death or withdrawal from the trial). A response was defined as a CR or PR, and no response was defined as SD or PD. For the clinical response rate and survival analyses, all patients (N=104) were included in the analyses. One patient was randomized twice but enrolled once based upon “human error”, so we cannot include all randomized patients (N=105). Of the 104 patients who received ICT, 94 patients had available tissue samples for correlation of biomarkers with clinical response (Fig. S4a).

We defined a modified BOR excluding surgery effect to correlate potential biomarkers to true biological responses without interference from cytoreductive surgery that removed target lesion(s) from some patients (Table S4). For patients (n=12) who had surgery removing target lesion(s), BOR excluding surgery effect was based on assessment of the remaining target lesions with exclusion of resected target lesion(s). For those patients (n=4) with only one metastatic lesion resected by metastasectomy, BOR was based upon the clinical response before surgery. Disease control was defined as a CR, PR or SD. Overall survival was defined as number of months from randomization to death (event) or last contact for patients who were alive at the final data collection. PFS was defined as the time from randomization until progression or death, whichever came first (event), or last follow-up for disease assessment among patients who were alive and free of disease at the last assessment. Estimates of OS and PFS (including those in *ad hoc* analyses of patients who received ICT plus surgery and patients who received only ICT without surgery) were calculated and graphed by Kaplan-Meier methods. Patient treatment allocation and tissue analysis is shown in Fig. S1.

### Whole Exome Sequencing

Tumor tissue samples and matched peripheral blood mononuclear cells (PBMCs, controls) from 42 patients were processed for whole exome sequencing. DNA from FFPE tissues and PBMCs was extracted using the QiaAmp DNA FFPE Tissue Kit and QiaAmp DNA Mini kit, respectively (Qiagen). Library construction was performed using manufacturer’s instructions. Briefly, ~250 ng genomic DNA was sheared using the Covaris S2 sonicator. KAPA Hyper Prep Kit with Agilent SureSelect XT Target Enrichment System was used for end repair, A-base addition, adaptor ligation, and library enrichment PCR. Sample concentrations were measured following library construction using the Agilent Tapestation. Hybridization reaction was then performed for exon capture using the manufacturer’s guidelines (Agilent SureSelect-XT Human All Exon v4). The libraries were normalized to equal concentrations using a QuantStudio 6 Flex instrument and pooled to equimolar amounts. Libraries were quantified using the Agilent Tapestation and sequenced using the Illumina HiSeq 2500 platform at a coverage of ~200X for tumor samples and ~100X for normal samples. The BWA aligner 40 was used for sequence alignment to the human reference genome, GRCh37 (UCSC genome browser: genome.ucsc.edu). The average exome- wide coverage ranges in 93.6–302.3-fold (median 200.7) in tumor samples and 54.1–294.3-fold (median 99.5) in the matched PBMC samples. SNV and indel calls were made with Mutect^30^ and Pindel^31^ respectively. The mutations were annotated by ANNOVAR^32^. Germline variants were filtered using germline DNA from paired blood samples. The resulting variants were filtered further by the following criteria to get the final variants: (a) dbSNPs that were “novel” and the ones already existing in COSMIC were included; (b) each variant had a coverage of at least 20x for tumor and 10x for normal samples; (c) SNVs with a VAF >=0.05 and <0.02 for tumor and normal samples, respectively and at least 3 reads to support the call at SNV in tumor sample were included; (d) for exclusion of common variants, only variants with AF<0.01 in Exome Aggregation Consortium (EXAC), ESP600 and 1000 Genome (1KG) were included; (e) only variants with LOD score >=6.3 (Mutect default) for tumor samples were included; (f) silent mutations, 5’UTR and 3’UTR mutations were excluded. Tumor mutational burden (TMB) was calculated based on counts of somatic mutations per Mb of captured region. Neoantigen prediction was performed using NetMHCpan (http://www.cbs.dtu.dk/services/NetMHCpan/). Briefly, all possible 8 to 12-mer peptides containing the mutated amino acid were used in the prediction. A binding affinity of less than 500 nM was used as cut off of predicted neoantigen for each nonsynonymous mutation. Statistical significance was calculated using Wilcoxon rank sum test to compare TMB or neoantigen load between groups. P < 0.05 was considered statistically significant. Copy number alterations (CNAs) were identified using in-house algorithm as previously described^33^. In brief, the copy number log2 ratios of tumor versus matched normal were calculated across the entire capture regions and then subjected to segmentation using circular binary segmentation (CBS)^34^. A cutoff of log2 ratio <= −0.325 was applied to identify copy number loss and log2 ratio >=0.325 was applied for copy number gain. An oncoplot plot was generated using maftools^35^.

### NanoString analysis

Pre-treatment tumor tissue samples from 83 patients were processed for RNA isolation. FFPE tissues were subjected to de-waxing using deparaffinization solution (Qiagen, Valencia, CA) prior to RNA isolation. Total RNA was extracted using the RecoverALL^™^ Total Nucleic Acid Isolation kit (Ambion, Austin, TX) for FFPE tissues and RiboPure^™^ RNA Purification Kit (Thermo Fisher Scientific) for fresh-frozen tissues according to the manufacturer’s instructions. Extracted RNA was quantified by ND Nanodrop1000 spectrometer (Thermo Scientific, Wilmington, MA, USA). For NanoString assay, 100 ng of RNA was used to detect immune gene expression using nCounter PanCancer Immune Profiling panel along with custom CodeSet. Counts of the reporter probes were tabulated for each sample by the nCounter Digital Analyzer and raw data output was imported into nSolver (http://www.nanostring.com/products/nSolver, v4.0) for normalization. Negative controls were subtracted as a background correction. Positive controls and housekeeping genes were used for normalization with the default parameters. Batch effect was corrected using the R *sva* package^36^. A 24-gene TLS signature was derived using a candidate gene approach and genes were selected based on two criteria: (i) biological relevance and (ii) gene sets from published studies of TLS^37, 38, 39, 40, 41^. Z scores were computed for TLS signature, IFN-γ signature^10^. Briefly, for gene expression signature analysis, the z score standardized values of each member gene in the gene set was averaged into a combined z score by using the square root of the number of member genes as the denominator to stabilize the variance of the mean. The list of genes for the TLS and IFN-γ signatures can be found in Table S7. Statistical significance was calculated using Welch’s t-test to compare Z scores between two groups and using Welch’s ANOVA to compare Z scores across three or more groups. Pairwise t-test was used to compare matched pre- treatment and post-treatment samples for signature Z scores and gene expression. Benjamini- Hochberg correction was applied for multiple tests. Patients were segregated into TLS^high^ and TLS^low^ group based on the median of TLS z score. Similarly, patients were segregated into IFNG^high^ and IFNG^low^ group based on the median of IFN-γ z score. Statistical significance was calculated using Fisher’s exact test to compare patient counts in different groups. P < 0.05 was considered statistically significant.

### CODEX

Codex staining assays were carried out according to the manufactured protocol. Briefly, 4 μm FFPE tissue section of were placed on poly-L-lysine coated coverslip (22mm × 22 mm). Section were stored at 4°C until use. Purified antibodies were obtained from the listed vendors (Table S8). Barcode and reporters were purchased form Akoya Bioscience (Table S8). Antibodies conjugated for CD107A, CD11C, CD20, CD21, CD31, CD44, CD45RO, CD68, CD8, ECADHERIN,KI67 and PANCYTOKERATIN were tagged with CODEX^®^ Barcodes at purchase (Akoya Biosciences). For CD15, CD23, CD3, CD4, CD47, EOMES, FOXP3, HLA-DR, ICOS, LAG3, MMP9, PD-1, and T-BET, 50ug of antibody (purified and free of BSA and glycerol) was conjugated in house following manufacture recommendations. Briefly, partially reduced antibodies were incubated with a unique DNA oligonucleotide (barcode), then barcode-conjugated antibody were purified using a 50-kDa centrifugal filter and collected with antibody storage solution. Purified barcoded-conjugated antibodies were stored at 4°C and used within 6 months of conjugation. . The antibody conjugation reactions were validated via protein gel electrophoresis. IHC and Codex staining were validated using human tonsil tissue. Antigen retrieval Tris–EDTA pH 9.0 was used during the staining protocol. Antibodies were incubated either O/N at 4°C or 3 hours at room temperature, depending on the optimization protocol (Table S8).

#### Codex imaging:

The regions of interest were determined using IHC and mIF staining and were representative of the microenvironment of the tissue sample. Stained sections were capture using a Keyence BZ-X810 inverted microscope with filter cubes 4900-UF1 Dapi, 49011-UF1 Alexa Fluor 488, 49004-UF1 Cy3 and 49006-UF1 Cy5 for the detection the corresponding fluorescent reporter. Exposure times for each antibody is shown in Table S8. A region of interest of 2.754 × 2.065 mm (5×5 tiles) was capture using at 20x of magnification and Z-stack of pitch of 1.5 μm with 9 slices.

#### Image processing, segmentation and analysis:

Alignment of images across cycles, stitching of tiles and subtraction of auto-fluorescence was performed using CODEX^®^ Processor application. A neural network-based cell segmentation tool DeepCell^10^ was applied to pre-processed images on DAPI channels to identify nuclei, and these nuclear masks were dilated to obtain whole-cell segmented cells. Nuclear segmentation masks were stochastically dilated by flipping pixels with a probability equal to the fraction of already-assigned neighboring pixels, an algorithm that resembles a diffusion process: for rounds 1 to 9: for each nuclear mask “M”, for each pixel on the border of “M”, count the number of its neighbor pixels that are already assigned, compute the “p”, the fraction of neighbor pixels already assigned to cells with probability “p” add the pixel to mask “M”. The dilation was done 9 times to obtain cell masks approximating cell shapes.

#### CODEX data analysis:

All mIF analyses were run in R-4.0.5 unless otherwise indicated. R functions are specified using the following notation:“<package_name>::<function_name>”

##### Cell clustering:

For cell clustering and cell neighborhood analysis, data from 8 cases (PD=4, PR=4) were combined using a total of twenty-five ROIs to include associated tissue heterogeneity. Possible batch effects were addressed by performing an inverse hyperbolic sine transform (“base::asinh”) on cell expression values for every marker, in every ROI. The normalized values were z-scaled across both cells and markers. To cluster cells, dimensionality reduction was first performed on scaled expression values using principal component analysis with 20 components (“stats::prcomp”). Next, a k-nearest neighbor graph was constructed to build a similarity network between cells in principal component space (“dbscan::kNN”, k = 30). Finally, cells were clustered using the Leiden graph clustering algorithm (“igraph::cluster_leiden”, cluster_resolution = 1.0). To label clusters, a heatmap showing the average normalized marker expression in each cluster was plotted. Clusters were annotated using their average expression to identify cell types, and these annotations were validated by manual inspection of multiplexed immunostains on images.

##### Construction of a spatial cellular graph:

To perform cell to cell interaction, a spatial graph of nearest neighbor was first constructed. Cell coordinates were derived by taking the centroid of each segmented cell nucleus relative to the corner of the ROI. A k-nearest neighbor algorithm was next used on these coordinates (“dbscan::kNN”, k = 10). This graph thus represents, for each cell, its 10 closest neighbors in 2D space.

##### Cell distance analysis:

An algorithm was computed to determine cell distance for a given cell type pair and the average minimum distance was calculated. The algorithm proceeds as follows: For each cell *i* of cell type *A*, compute distances to all cells of type *B* (rdist::cdist); (2) for each *i,* compute the shortest distance to a cell of type *B* (the minimum distance); and computethe average of all cells *i* in each ROI. Finally, to compare this metric between patient cohorts, we performed a Welch Two Sample t-test on the average minimum distance metric between our two patient cohorts.

##### Cell neighborhood analysis:

To define cellular neighborhoods (CNs), the number of neighbors of each cell type was counted, resulting in a matrix of cells by cell clusters, with each row representing a cell, each column representing a cell annotation (cell type) from the clustering above, and each value representing the count of neighbors of the given annotation. The neighbor cell proportion was computed for each row. The resulting matrix was clustered using k-means clustering (“stats::kmeans”), where the optimal k was determined empirically by maximizing the silhouette score metric (“cluster::silhouette”). Each cluster was defined as a CN. Thus, each cell was given both a cell type annotation, which depends only on the cell’s own marker expression, and a cell neighborhood annotation, which depends on the cell’s type and the identities of its nearest neighbors. To compare CNs between patient cohorts, we determined the proportion of cells in each ROI belonging to each CN. Proportions were transformed using the inverse hyperbolic tangent (“base::asinh”) and split by cohort. We then performed pairwise t-tests (“stat::t.test”) on the transformed proportions, comparing each CN between PR and PD cohorts. The resulting p- values were corrected for multiple testing by the Bonferroni method (“stat::p.adjust”, method = “Bonferroni”).

#### Single-Cell RNA Sequencing:

sc-RNA-seq was performed using the 10x Genomics Chromium Single Cell Controller. Briefly, single-cell suspensions were prepared from PBMCs. Cells were resuspended in freezing media containing 90% AB serum (derived from donors with AB blood type) and 10% dimethyl sulfoxide (DMSO) and stored in liquid nitrogen until analysis. For sc-RNA-seq analysis, cells were thawed, washed, and droplet-separated using the Chromium Single Cell 5′ v.2 Reagent Kit (10X Genomics) with the 10x Genomics microfluidic system creating cDNA library with individual barcodes for individual cells. Barcoded cDNA transcripts from patients were pooled and sequenced using the NovaSeq 6000 Sequencing System (Illumina).

#### Single-Cell RNA sequencing analysis:

Raw sc-RNA-seq reads generated by Illumina sequencer were demultiplexed into FASTQ and aligned to GRCh38 reference genome to generate count matrices using Cell Ranger v7.1.0 analysis pipelines (10x Genomics). Potential doublets were removed with the DoubleFinder R package (v2.0.3)^42^. The Seurat R package (v4.0.3)^43^ was used to perform the analysis including filtering out low-quality cells, normalizing the data and clustering the cells. Briefly, genes presented in less 10 cells and cells with less than 500 genes or more than 5000 genes, or with more than 20% mitochondrial gene counts were excluded from downstream analysis. A global-scaling normalization method “LogNormalize” was applied to the raw expression (“Seurat::NormalizeData”) with the default scale factor (10000). The top 2000 highly variable genes were found with the “vst” method (“Seurat::FindVariableFeatures”) and their normalized expression was scaled (“Seurat::ScaleData”) with regression out library size and cell cycle effects. Principal component analysis (PCA) was performed with the highly variable genes (“Seurat::RunPCA”) and Harmony R package (v0.1.0)^44^ was used to integrate the data sets with the first 50 PCA components (“harmony::RunHarmony”). Then the first 30 components from harmony were used for constructing KNN (K-nearest neighbor) and SNN (shared nearest neighbor) graphs (“Seurat::FindNeighbors”). Cells were clustered with Louvain algorithm based on the SNN graph (“Seurat::FindClusters”) at resolution 0.4. UMAP projection was performed (“Seurat:: RunUMAP”) with parameters (reduction = “harmony”, dims = 1:30, n.neighbors = 20, min.dist = 0.2, spread = 1). For T and NK cells subset analysis, we selected the T and NK cells and performed the similar data analysis described above with slightly different parameters (top 1000 variable genes, first 20 harmony components for building the graphs and UMAP projection, resolution 0.8 for clustering, min. dist 0.05 and spread 2 for UMAP projection) Principal component analysis (PCA) was applied to the top 2000 highly variable genes and Harmony R package (v0.1.0)^44^ was used to integrate the data sets with the first 50 PCA components. Then the first 30 components from harmony were used for constructing a KNN graph, clustering and UMAP projection.

## Supplementary Material

1

## Figures and Tables

**Table 1.** Grade 3 and higher treatment related adverse events.

Adverse Event	Total (N=104) Grade 3+		Nivo (N=29) Grade 3+		Nivo+Bev (N=45) Grade 3+		Nivo+Ipi (N=30) Grade 3+	
	n	(%)	n	(%)	n	(%)	n	(%)
**Any Grade 3+ Related Event**	**34**	**(33)**	**8**	**(28)**	**17**	**(38)**	**9**	**(30)**
Lipase increased	15	(14)	5	(17)	5	(11)	5	(17)
Hypertension	9	(9)	0	(0)	8	(18)	1	(3)
Amylase increased	6	(6)	1	(3)	2	(4)	3	(10)
ALT increase	3	(3)	1	(3)	1	(2)	1	(3)
Colitis	3	(3)	1	(3)	2	(4)	0	(0)
Pneumonitis	3	(3)	0	(0)	3	(7)	0	(0)
Anemia	2	(2)	1	(3)	1	(2)	0	(0)
Fatigue	2	(2)	0	(0)	2	(4)	0	(0)
Hyperglycemia	2	(2)	0	(0)	1	(2)	1	(3)
Lymphocyte decrease	2	(2)	0	(0)	1	(2)	1	(3)
Pain	2	(2)	0	(0)	1	(2)	1	(3)
Pancreatitis	2	(2)	2	(7)	0	(0)	0	(0)
AST increased	1	(1)	1	(3)	0	(0)	0	(0)
Adrenal insufficiency	1	(1)	1	(3)	0	(0)	0	(0)
Anorexia	1	(1)	0	(0)	1	(2)	0	(0)
Appendicitis	1	(1)	0	(0)	1	(2)	0	(0)
Aspiration	1	(1)	0	(0)	0	(0)	1	(3)
Atrial fibrillation	1	(1)	0	(0)	0	(0)	1	(3)
Bilirubin increase	1	(1)	1	(3)	0	(0)	0	(0)
C. difficile infection	1	(1)	0	(0)	1	(2)	0	(0)
Calcium pyrophosphate arthropathy	1	(1)	0	(0)	0	(0)	1	(3)
Dehydration	1	(1)	0	(0)	1	(2)	0	(0)
Diarrhea	1	(1)	0	(0)	1	(2)	0	(0)
Dyspnea	1	(1)	0	(0)	1	(2)	0	(0)
Glycosuria	1	(1)	0	(0)	0	(0)	1	(3)
Hemoptysis	1	(1)	0	(0)	1	(2)	0	(0)
Hypokalemia	1	(1)	0	(0)	1	(2)	0	(0)
Hypophosphatemia	1	(1)	1	(3)	0	(0)	0	(0)
Hypotension	1	(1)	0	(0)	1	(2)	0	(0)
Insulin deficiency	1	(1)	0	(0)	0	(0)	1	(3)
Joint range of motion decreased	1	(1)	0	(0)	1	(2)	0	(0)
Mucositis oral	1	(1)	0	(0)	1	(2)	0	(0)
Nausea	1	(1)	0	(0)	1	(2)	0	(0)
Proteinuria	1	(1)	0	(0)	1	(2)	0	(0)
Rash	1	(1)	0	(0)	1	(2)	0	(0)
Urinary tract infection	1	(1)	0	(0)	1	(2)	0	(0)

**Table 2.** Surgery details and complications.

Surgical Details	n=43
**ICT Therapy, n (%)**	
Nivolumab	14 (33)
Nivolumab + Bevacizumab	16 (37)
Nivolumab + Ipilimumab	13 (30)
**Surgery Type, n (%)**	
CN	39 (91)
Metastasectomy	4 (9)
CN details, n (%)*	
Open	23 (59)
Laparoscopic	16 (41)
Included thrombectomy	9 (23)
Included RPLND	18 (46)
**Median EBL, mL (IQR)**	200 (100–500)
**Intraoperative complications**	0
**90-day postoperative complications**	6 (14)
***Historical CN complication rates*** ^***Bhindi et al*.**^	*12–57%*

* Percent of cytoreductive nephrectomy patients (n=39)

CN: cytoreductive nephrectomy;

RPLND: retroperitoneal lymph node dissection;

EBL: estimated blood loss; IQR: interquartile range

## Data Availability

All whole exome sequencing, sc-RNA sequencing and gene expression data (NanoString) that support the findings of this study have been deposited in European Genome-phenome Archive (EGA) and are accessible through the EGA accession number EGAS00001005667. All other relevant data related to the current study are available from the corresponding author (Padmanee Sharma) on reasonable request that does not include confidential patient information.
