# Supplementary material for: Safety outcomes and immunological correlates in a prospective clinical trial of immune checkpoint therapy plus debulking surgery for patients with metastatic renal cell carcinoma
Source: Res Sq. 2024 Nov 12:rs.3.rs-4331053. Preprint. [Version 1] doi: 10.21203/rs.3.rs-4331053/v1 (PMC11601845; doi:10.21203/rs.3.rs-4331053/v1)
Supplement: 1 [file NIHPPrs4331053V1-supplement-1.pdf]

Figure S1a

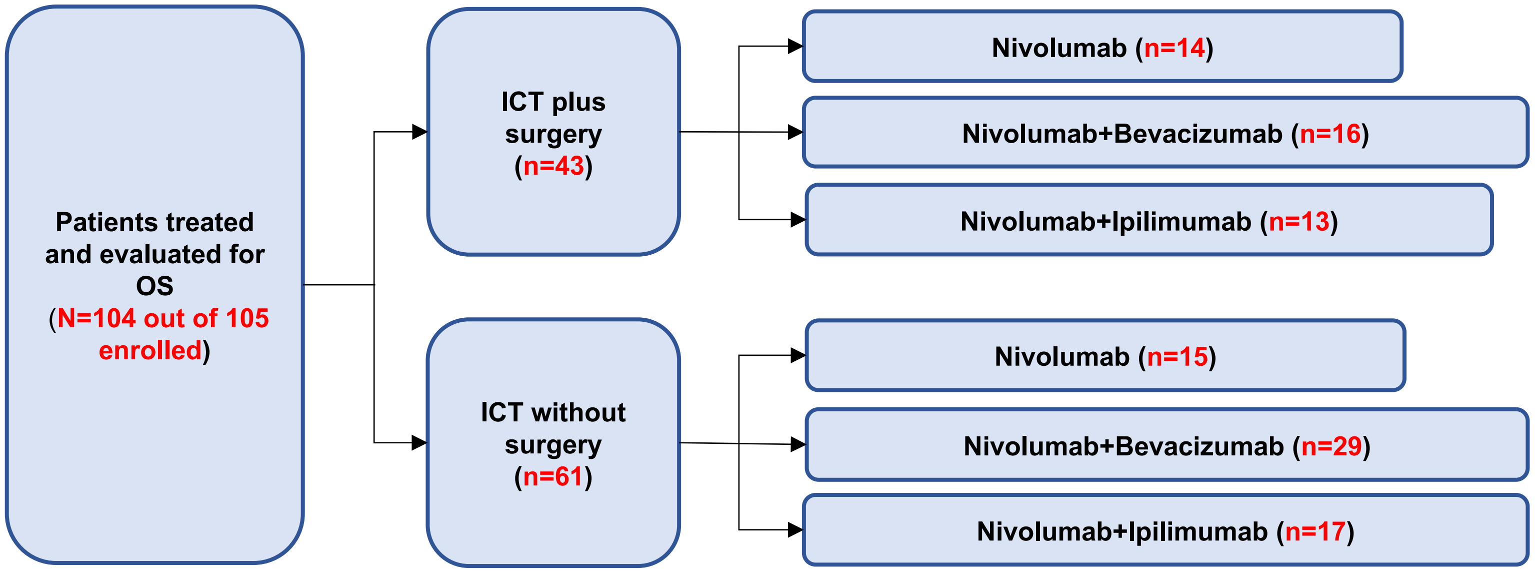
Patients treated and evaluated for OS (N=104 out of 105 enrolled)
ICT plus surgery (n=43)
ICT without surgery (n=61)
Nivolumab (n=14)
Nivolumab+Bevacizumab (n=16)
Nivolumab+Ipilimumab (n=13)
Nivolumab (n=15)
Nivolumab+Bevacizumab (n=29)
Nivolumab+Ipilimumab (n=17)

Figure S1b

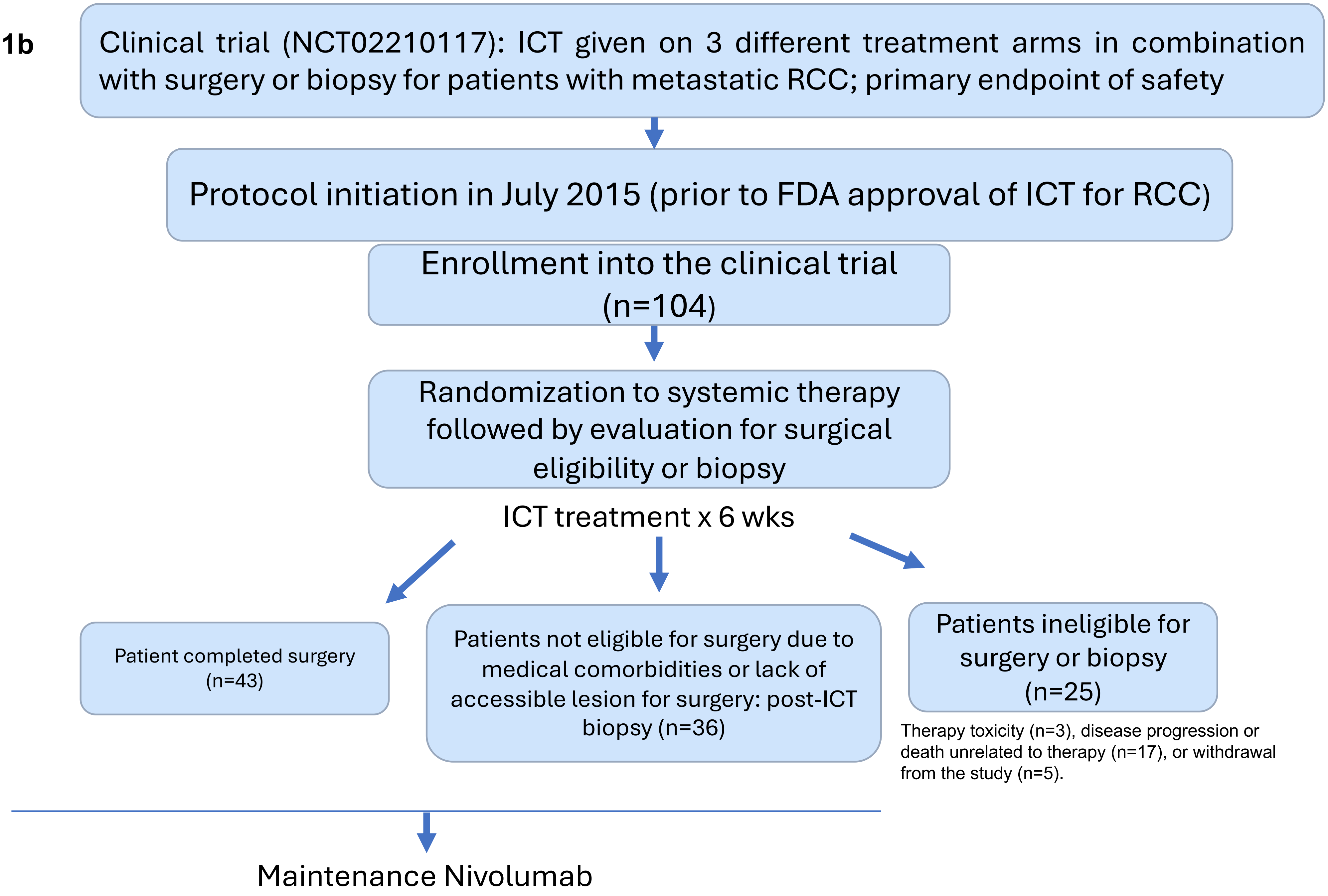
Clinical trial (NCT02210117): ICT given on 3 different treatment arms in combination with surgery or biopsy for patients with metastatic RCC; primary endpoint of safety
Protocol initiation in July 2015 (prior to FDA approval of ICT for RCC)
Enrollment into the clinical trial (n=104)
Randomization to systemic therapy followed by evaluation for surgical eligibility or biopsy
ICT treatment x 6 wks
Patient completed surgery (n=43)
Patients not eligible for surgery due to medical comorbidities or lack of accessible lesion for surgery: post-ICT biopsy (n=36)
Patients ineligible for surgery or biopsy (n=25)
Therapy toxicity (n=3), disease progression or death unrelated to therapy (n=17), or withdrawal from the study (n=5).
Maintenance Nivolumab

Fig. S1. (a) Diagram of the patients treated with ICT plus surgery (n=43) or ICT plus biopsy (n=36) or ICT without either surgery or biopsy (n=25). Of the 105 patients enrolled and randomized to ICT, 104 patients received therapy and were evaluated for clinical outcomes. (b) Flow chart depicting the distribution of patients (N=104) into surgery (n=43), biopsy (n=36) and no procedure (n=25).

Figure S2

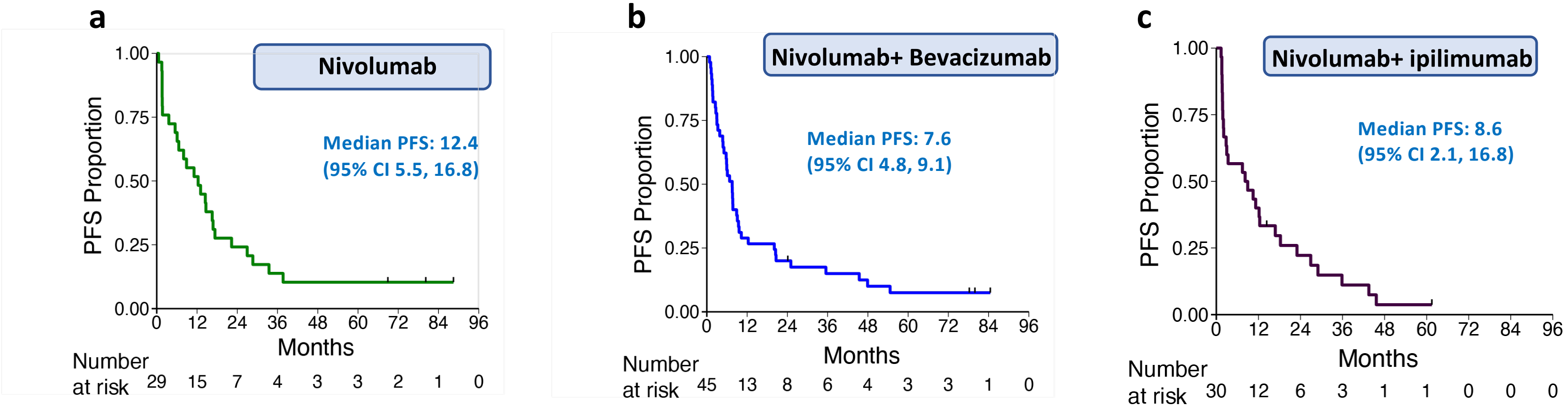

Fig. S2. (a) PFS in Arm A (nivolumab); (b) PFS in Arm B (nivolumab + bevacizumab); (c) PFS in Arm C (nivolumab + ipilimumab)

Figure S3

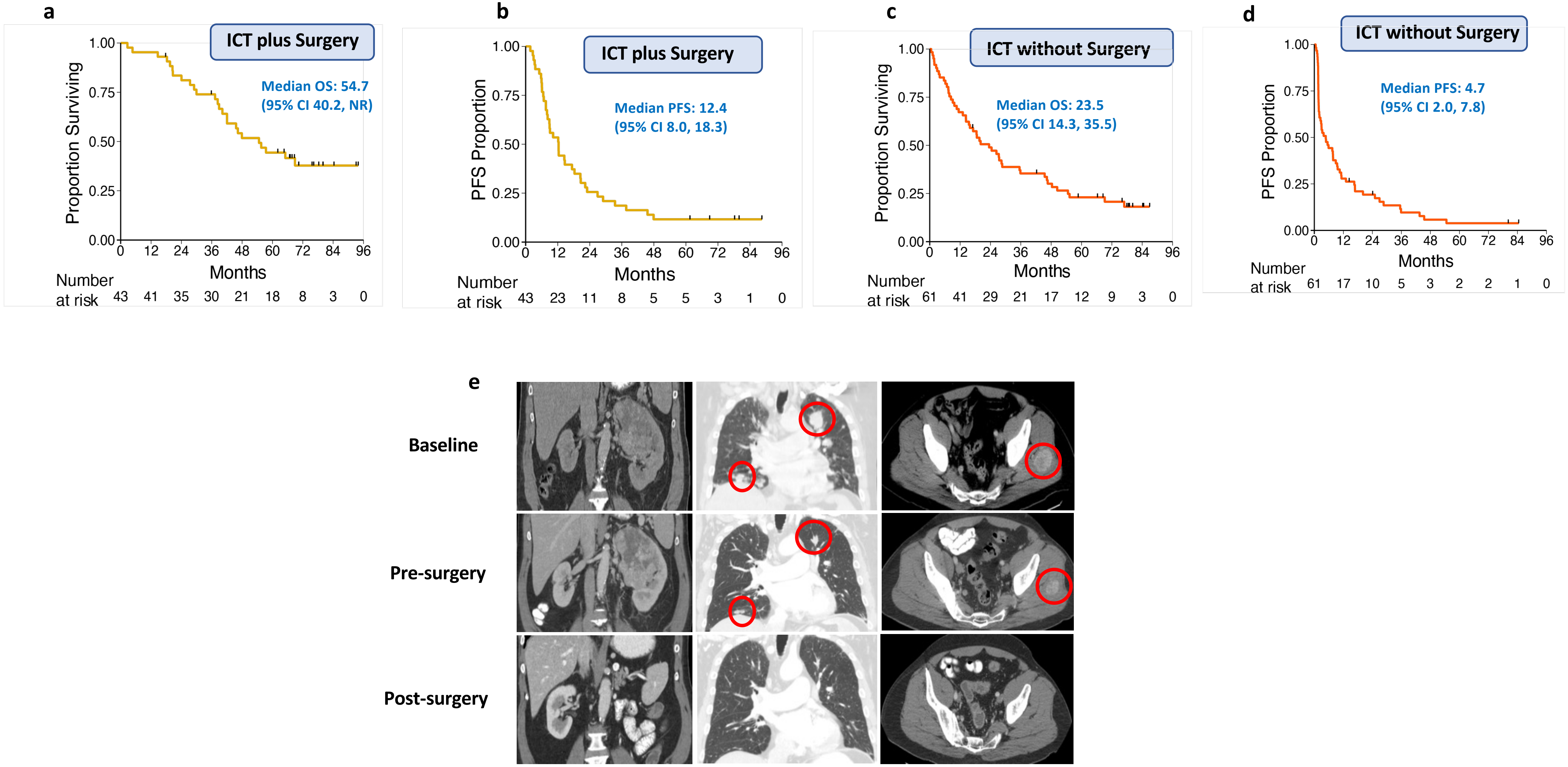
a
ICT plus Surgery
Median OS: 54.7
(95% CI 40.2, NR)
Proportion Surviving
1.00
0.75
0.50
0.25
0.00
0 12 24 36 48 60 72 84 96
Months
Number at risk 43 41 35 30 21 18 8 3 0
b
ICT plus Surgery
Median PFS: 12.4
(95% CI 8.0, 18.3)
PFS Proportion
1.00
0.75
0.50
0.25
0.00
0 12 24 36 48 60 72 84 96
Months
Number at risk 43 23 11 8 5 5 3 1 0
c
ICT without Surgery
Median OS: 23.5
(95% CI 14.3, 35.5)
Proportion Surviving
1.00
0.75
0.50
0.25
0.00
0 12 24 36 48 60 72 84 96
Months
Number at risk 61 41 29 21 17 12 9 3 0
d
ICT without Surgery
Median PFS: 4.7
(95% CI 2.0, 7.8)
PFS Proportion
1.00
0.75
0.50
0.25
0.00
0 12 24 36 48 60 72 84 96
Months
Number at risk 61 17 10 5 3 2 2 1 0
e
Baseline
Pre-surgery
Post-surgery

Fig. S3. (a) OS for patients who had ICT plus surgery. NR: Not reached. (b) PFS for ICT plus surgery patients; (c) OS for patients who had ICT without surgery patients. (d) PFS for ICT without surgery patients; (e) Representative images from a patient who received nivolumab plus surgery. The top row demonstrates baseline images of left renal mass, lung and muscle metastases (circled). The middle row demonstrates response in metastatic lesions (circled) after 6 weeks of ICT but prior to surgery (pre-surgery). The bottom row demonstrates response in metastatic lesions after ICT and 7 months after surgery, indicating absence of recurrence and resolution of lung and muscle metastases (post-surgery).

Figure S4 a

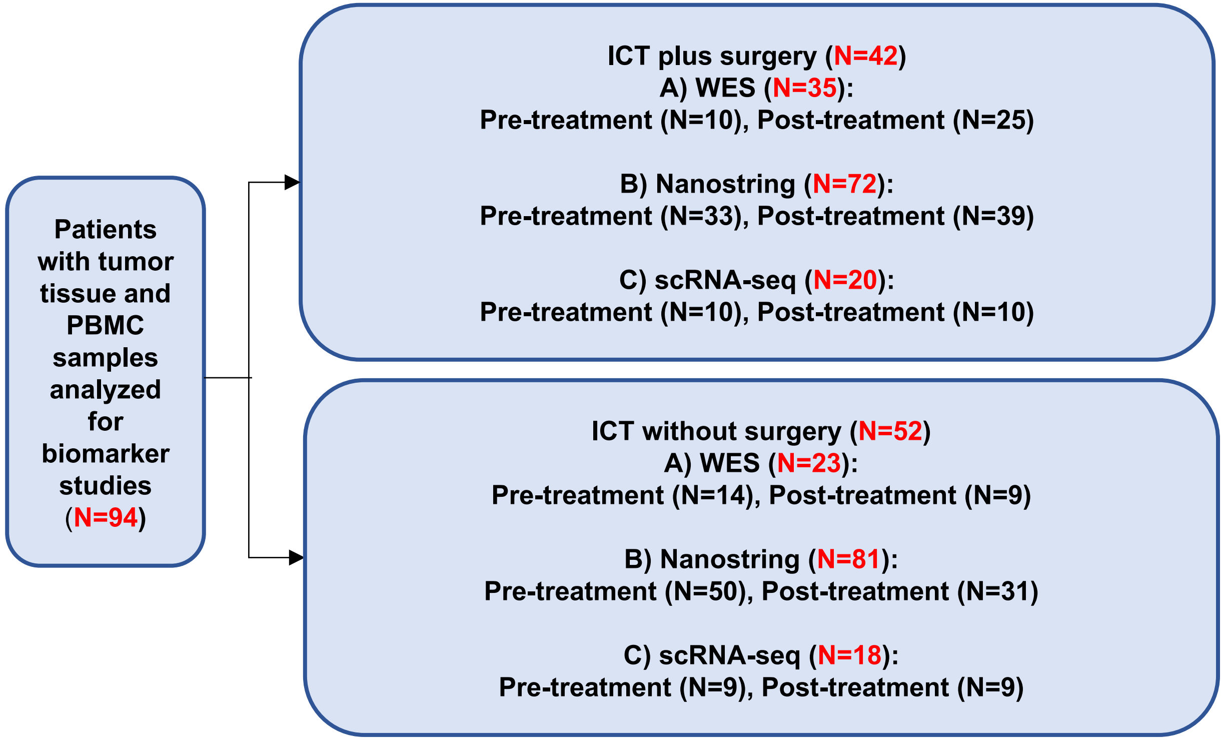
Patients with tumor tissue and PBMC samples analyzed for biomarker studies (N=94)
ICT plus surgery (N=42)
A) WES (N=35):
Pre-treatment (N=10), Post-treatment (N=25)
B) Nanostring (N=72):
Pre-treatment (N=33), Post-treatment (N=39)
C) scRNA-seq (N=20):
Pre-treatment (N=10), Post-treatment (N=10)
ICT without surgery (N=52)
A) WES (N=23):
Pre-treatment (N=14), Post-treatment (N=9)
B) Nanostring (N=81):
Pre-treatment (N=50), Post-treatment (N=31)
C) scRNA-seq (N=18):
Pre-treatment (N=9), Post-treatment (N=9)

Figure S4

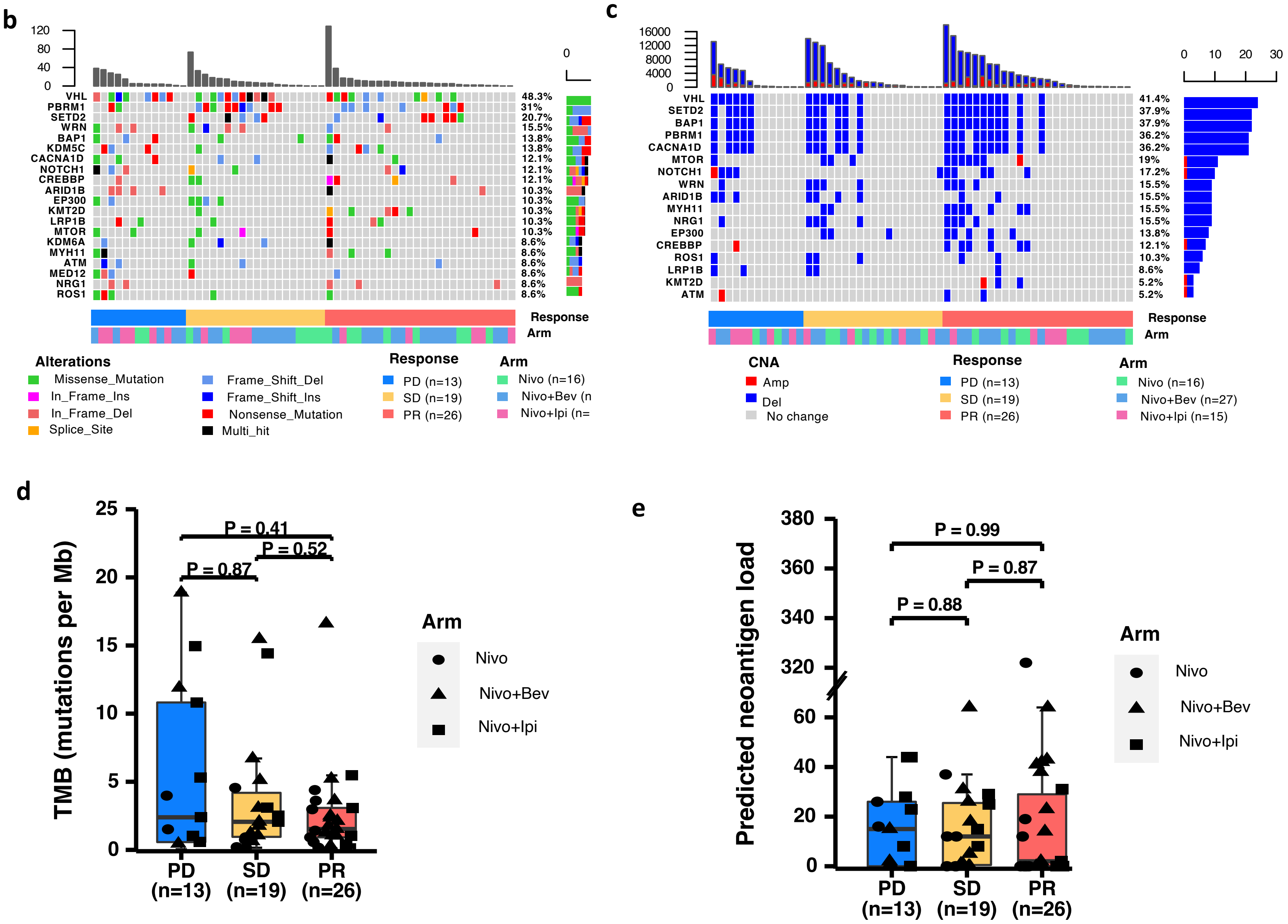
b
VHL
PBRM1
SETD2
WRN
BAP1
KDM5C
CACNA1D
NOTCH1
CREBBP
ARID1B
EP300
KMT2D
LRP1B
MTOR
KDM6A
MYH11
ATM
MED12
NRG1
ROS1
48.3%
31%
20.7%
15.5%
13.8%
13.8%
12.1%
12.1%
12.1%
10.3%
10.3%
10.3%
10.3%
10.3%
8.6%
8.6%
8.6%
8.6%
8.6%
8.6%
Response
Arm
Alterations
Missense_Mutation
In_Frame_Ins
In_Frame_Del
Splice_Site
Frame_Shift_Del
Frame_Shift_Ins
Nonsense_Mutation
Multi_hit
Response
PD (n=13)
SD (n=19)
PR (n=26)
Arm
Nivo (n=16)
Nivo+Bev (n
Nivo+Ipi (n=
c
VHL
SETD2
BAP1
PBRM1
CACNA1D
MTOR
NOTCH1
WRN
ARID1B
MYH11
NRG1
EP300
CREBBP
ROS1
LRP1B
KMT2D
ATM
41.4%
37.9%
37.9%
36.2%
36.2%
19%
17.2%
15.5%
15.5%
15.5%
15.5%
13.8%
12.1%
10.3%
8.6%
5.2%
5.2%
Response
Arm
CNA
Amp
Del
No change
Response
PD (n=13)
SD (n=19)
PR (n=26)
Arm
Nivo (n=16)
Nivo+Bev (n=27)
Nivo+Ipi (n=15)
d
TMB (mutations per Mb)
P = 0.41
P = 0.52
P = 0.87
PD (n=13)
SD (n=19)
PR (n=26)
Arm
Nivo
Nivo+Bev
Nivo+Ipi
e
Predicted neoantigen load
P = 0.99
P = 0.87
P = 0.88
PD (n=13)
SD (n=19)
PR (n=26)
Arm
Nivo
Nivo+Bev
Nivo+Ipi

Fig. S4. (a) Of the 104 patients who received ICT, 93 patients had available tissue samples for correlation of biomarkers with clinical response. Available tumor samples that were of sufficient quality for whole exome sequencing (WES), NanoString and sc-RNA-seq analysis are listed for each group. (b) Oncoplot expanding on Fig. 2A showing somatic mutation landscape of top 20 most frequently mutated genes. Specific symbols and color schemes are exactly the same as those in Fig. 2a. (c) Analysis of Copy Number Alterations (CNA) in top 20 most commonly mutated genes in pre-treatment tumor samples with clinical response (n=58). Total percentage of CNA in each gene was denoted on the right. (d-e) Box plot showing association of tumor mutational burden (TMB) (d) and predicted neoantigen burden (e) with clinical responses (n=58). $p < 0.05$ was considered statistically significant. PD: progressive disease, SD: stable disease, PR: partial response. The following color scheme is used in all figures showing biological response groups; PD=blue, SD=yellow, and PR=red.

Figure S5

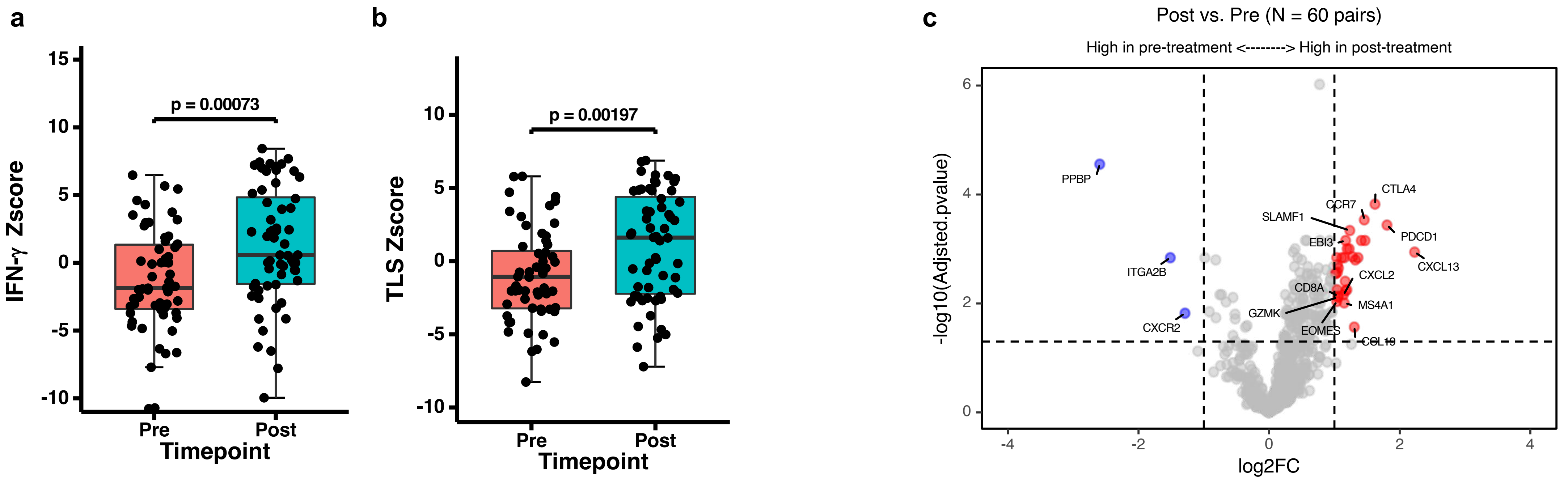
a
IFN-γ Zscore
p = 0.00073
Pre
Post
Timepoint
b
TLS Zscore
p = 0.00197
Pre
Post
Timepoint
c
Post vs. Pre (N = 60 pairs)
High in pre-treatment <--------> High in post-treatment
-log10(Adjsted.pvalue)
log2FC
PPBP
ITGA2B
CXCR2
CTLA4
CCR7
SLAMF1
PDCD1
EBI3
CXCL13
CXCL2
CD8A
MS4A1
GZMK
EOMES
CCL19

Figure S5

d

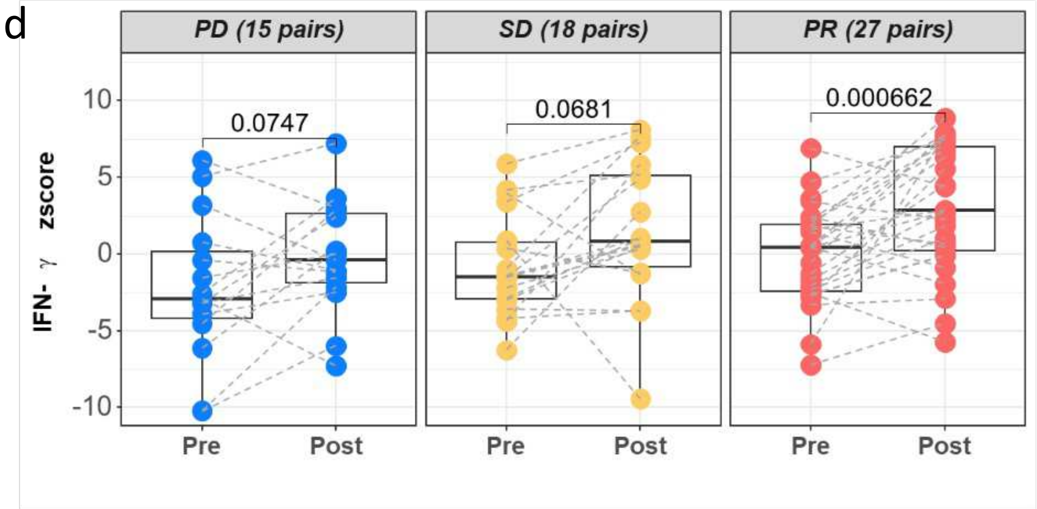
PD (15 pairs)
SD (18 pairs)
PR (27 pairs)
0.0747
0.0681
0.000662
IFN-γ zscore
10
5
0
-5
-10
Pre
Post
Pre
Post
Pre
Post

e

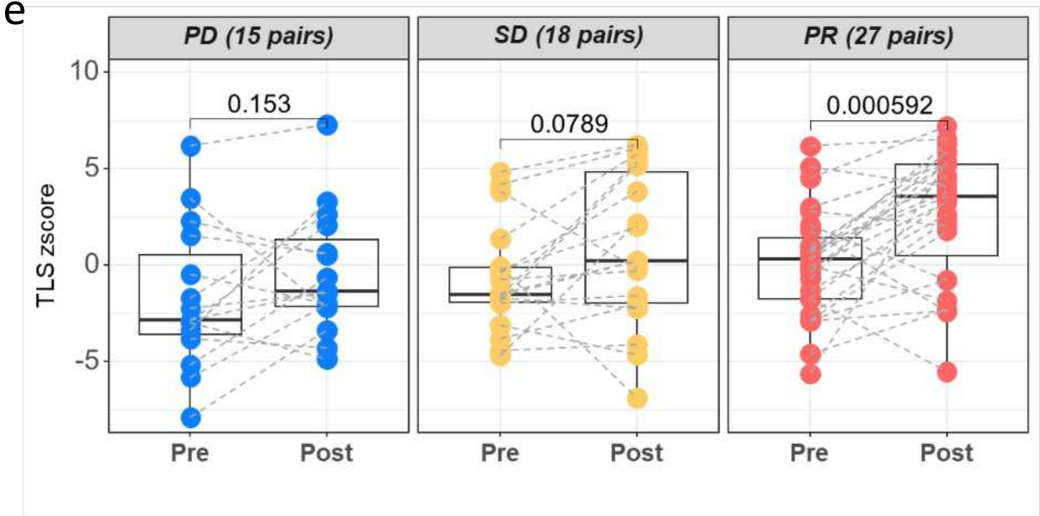
PD (15 pairs)
SD (18 pairs)
PR (27 pairs)
0.153
0.0789
0.000592
TLS zscore
10
5
0
-5
Pre
Post
Pre
Post
Pre
Post

Fig. S5. **NanoString gene expression analysis of matched pre-treatment and post-treatment tumor samples (n=60).**

Boxplot showing IFN-gamma z score ($p=0.00073$) (a) and TLS z score ($p=0.00197$) (b) in matched pre-treatment and post-treatment tumor samples. (c) A volcano plot showing the DEG analysis of paired pre- and post-treatment samples (n=60 pairs). Horizontal dashed line indicates FDR=0.05. Vertical dashed lines: $\log2(FC) > 1$ (right) or < -1 (left). Pairwise t-test was performed. $p<0.05$ indicate statistical significance. NanoString gene expression analysis of matched pre-treatment and post-treatment tumor samples from response groups. Boxplot showing IFN-gamma z score (d) and TLS z score (e) in matched pre-treatment and post-treatment tumor samples from different response groups.

Figure S6

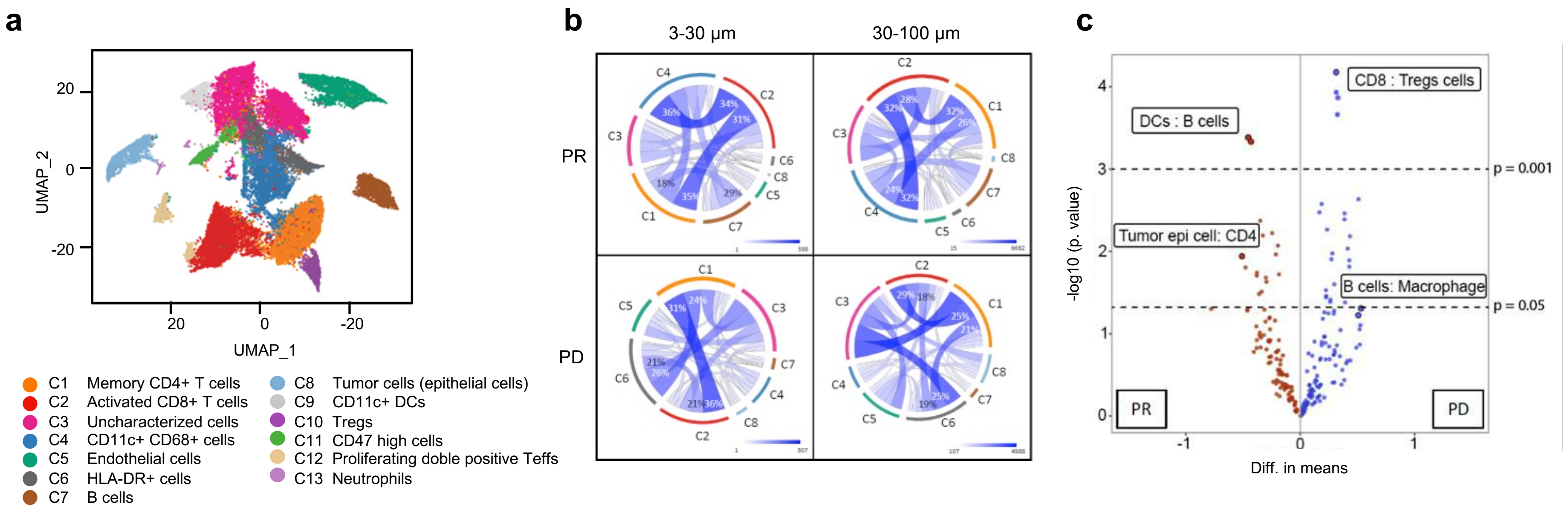

Fig.S6. (a) To assess cell-cell interaction, clustering of one pair of PR and PD generated 13 clusters (b) Circle plots for cell-cell interactions between 3-30 microns and 30-100 microns between cell types are depicted, focusing only on major clusters (C1 to C8) for clarity. The width of the ribbons represents the number of interactions, and the percentage of interactions within each cluster is shown. The arc length is proportional to cluster size, and color-coding is based on cluster identification in Figure S6a. Interactions between CD4, CD8 T cells, and B cells are observed in the PR case. (c)Volcano plot comparing cell clusters between PR (n=4) and PD (n=4) cases, horizontal punctuated lines show significant levels p=0.001 and p=0.05. Differences in cell-cell interactions between tumor cells, B cell, DC, CD4, and CD8 T cell clusters are indicated.

Figure S7

Average distance to the nearest CD8 T cell

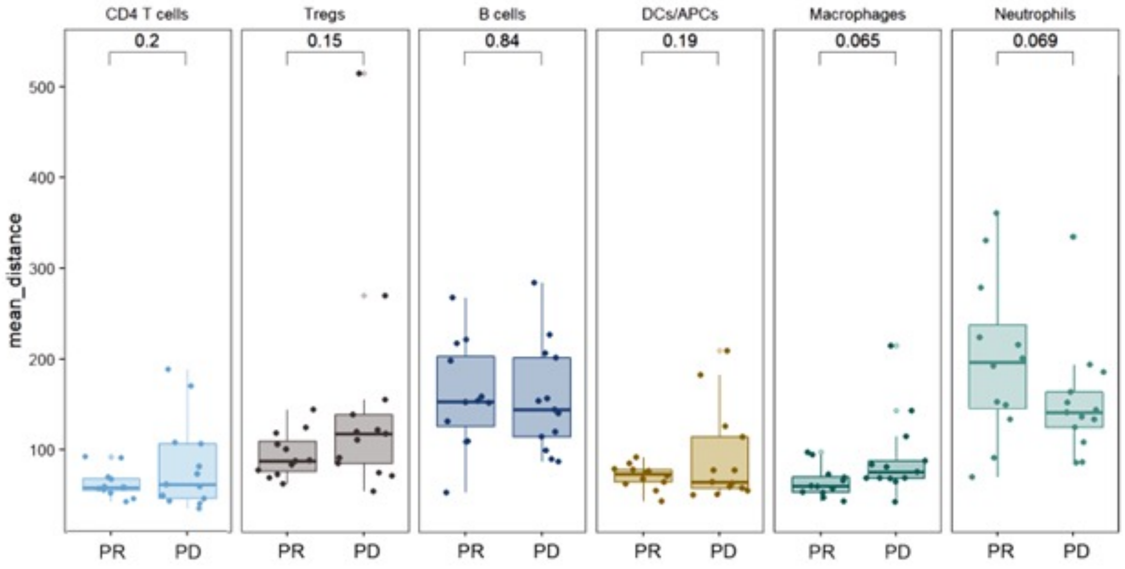

Average distance to the nearest Macrophage

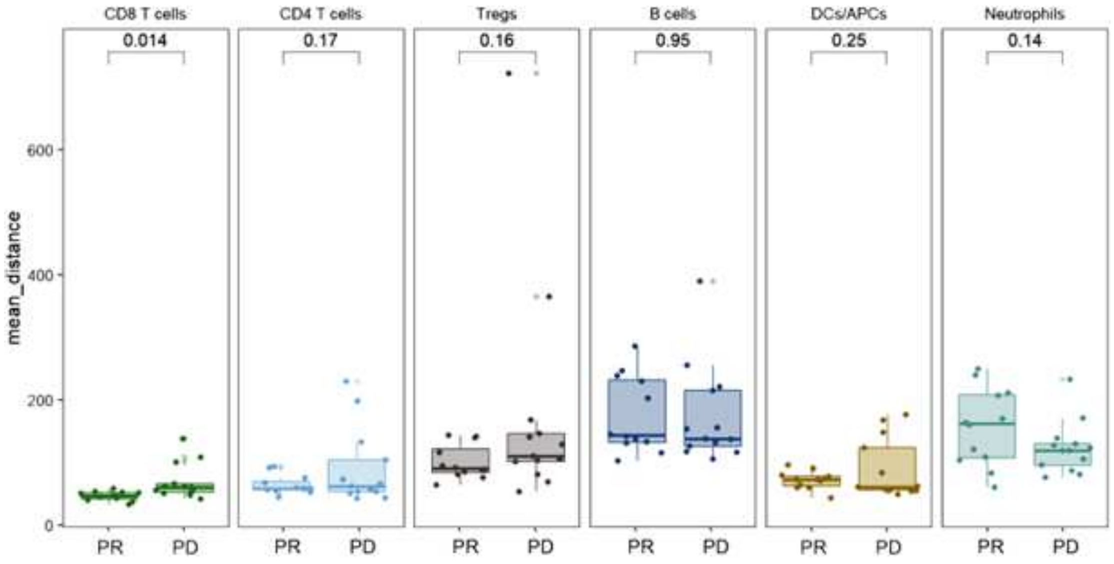

Average distance to the nearest CD4 T cell

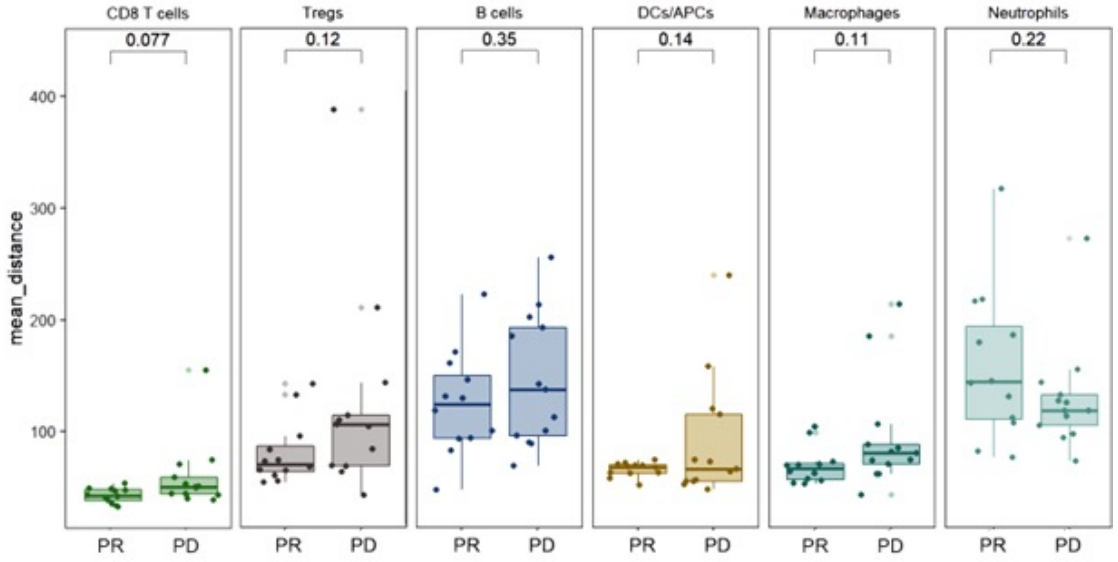

Fig. S7. Cell-cell distance analysis between different cell subsets is shown when comparing 25 regions of interest from a total of eight cases (PR=4, PD=4).

Figure S8

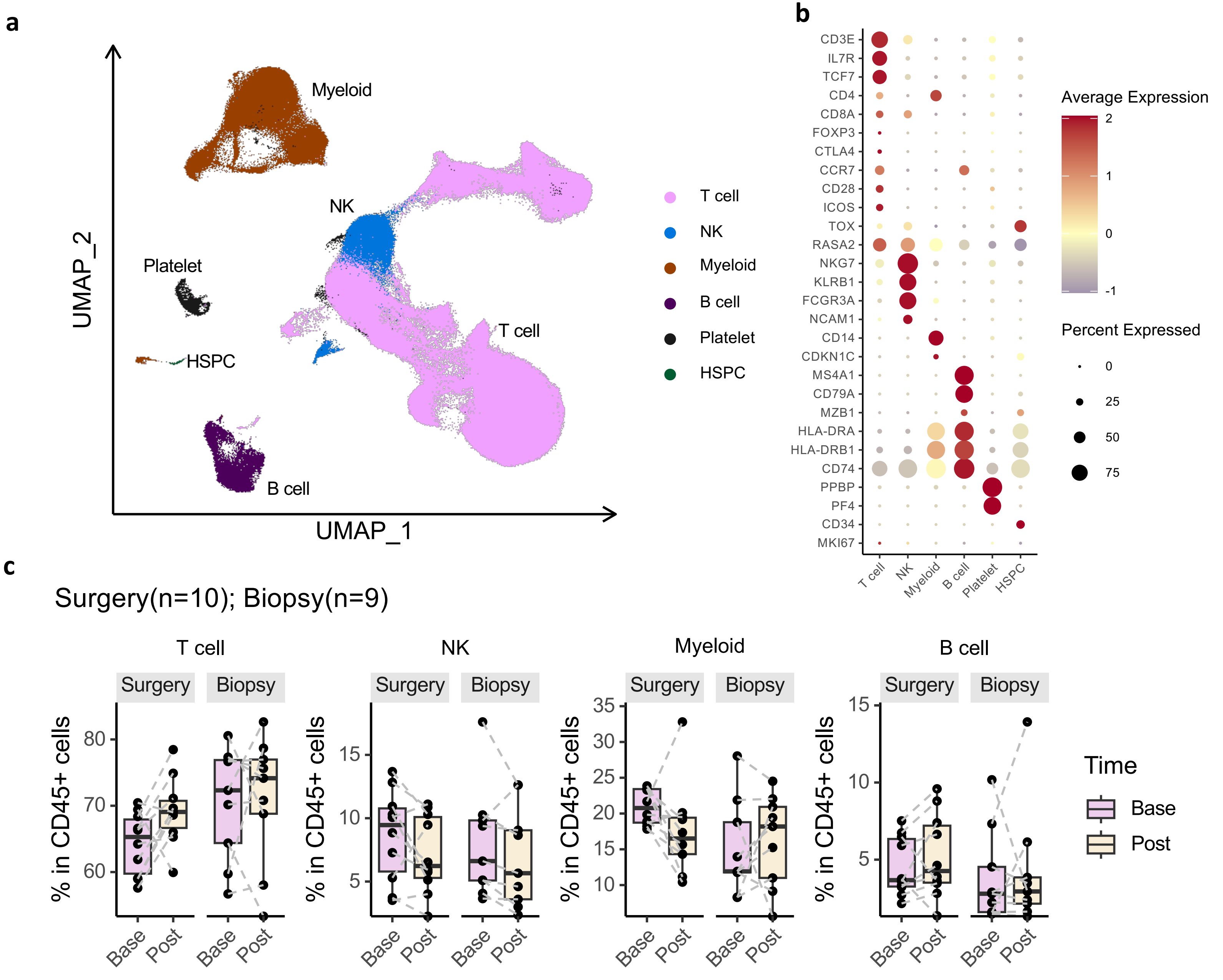
a
Myeloid
NK
Platelet
HSPC
T cell
B cell
UMAP_1
UMAP_2
T cell
NK
Myeloid
B cell
Platelet
HSPC
b
CD3E
IL7R
TCF7
CD4
CD8A
FOXP3
CTLA4
CCR7
CD28
ICOS
TOX
RASA2
NKG7
KLRB1
FCGR3A
NCAM1
CD14
CDKN1C
MS4A1
CD79A
MZB1
HLA-DRA
HLA-DRB1
CD74
PPBP
PF4
CD34
MKI67
T cell
NK
Myeloid
B cell
Platelet
HSPC
Average Expression
2
1
0
-1
Percent Expressed
0
25
50
75
c
Surgery(n=10); Biopsy(n=9)
T cell
NK
Myeloid
B cell
Surgery
Biopsy
% in CD45+ cells
Base
Post
Time
Base
Post

Fig. S8. **Sc-RNA-seq analysis of PBMC from patients.**

(a) UMAP plot of sc-RNA seq data depicting the different immune cell subsets (CD45+ cells) in the PBMC of patients. (b) Dotplot showing the average expression of indicated genes as well as the percentage of cells expressing the gene in the indicated immune cell populations. (c) Box plot of the frequency of immune cell populations at the indicated time point from patients who underwent surgery (n = 10) or biopsy (n = 9). BL: baseline.

Figure S9

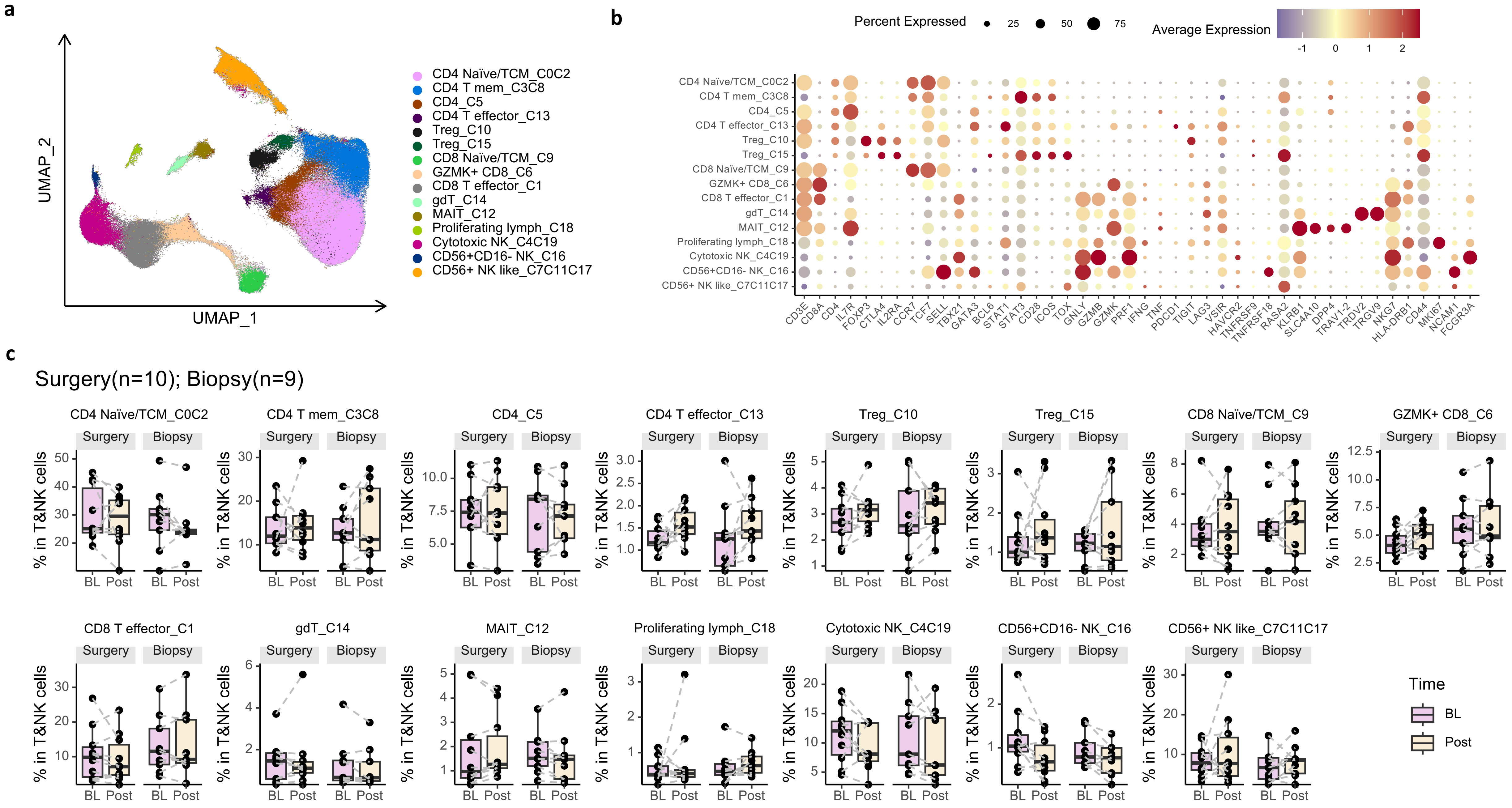
a
UMAP_2
UMAP_1
CD4 Naïve/TCM_C0C2
CD4 T mem_C3C8
CD4_C5
CD4 T effector_C13
Treg_C10
Treg_C15
CD8 Naïve/TCM_C9
GZMK+ CD8_C6
CD8 T effector_C1
gdT_C14
MAIT_C12
Proliferating lymph_C18
Cytotoxic NK_C4C19
CD56+CD16- NK_C16
CD56+ NK like_C7C11C17
b
Percent Expressed
25
50
75
Average Expression
-1
0
1
2
CD3E
CD8A
CD4
IL7R
FOXP3
CTLA4
IL2RA
CCR7
TCF7
SELL
TBX21
GATA3
BCL6
STAT1
STAT3
CD28
ICOS
TOX
GNLY
GZMB
GZMK
PRF1
IFNG
TNF
PDCD1
TIGIT
LAG3
VSIR
HAVCR2
TNFRSF9
TNFRSF18
RASA2
KLRB1
SLC4A10
DPP4
TRAV1-2
TRDV2
TRGV9
NKG7
HLA-DRB1
CD44
MKI67
NCAM1
FCGR3A
c
Surgery(n=10); Biopsy(n=9)
Surgery
Biopsy
% in T&NK cells
BL
Post
Time
BL
Post

Fig. S9. **Sc-RNA-seq sub-cluster analysis of T and NK cells from patients.**

(a) UMAP plot of sc-RNA-seq data depicting the different T and NK subsets in the PBMC of patients. (b) Dotplot showing the average expression of indicated genes as well as the percentage of cells expressing the gene in the indicated T and NK cell sub-populations. (c) Box plot of the frequency of T and NK cell sub-populations at the indicated time point from patients who underwent surgery (n = 10) or biopsy (n = 9). BL: baseline.

Figure S10

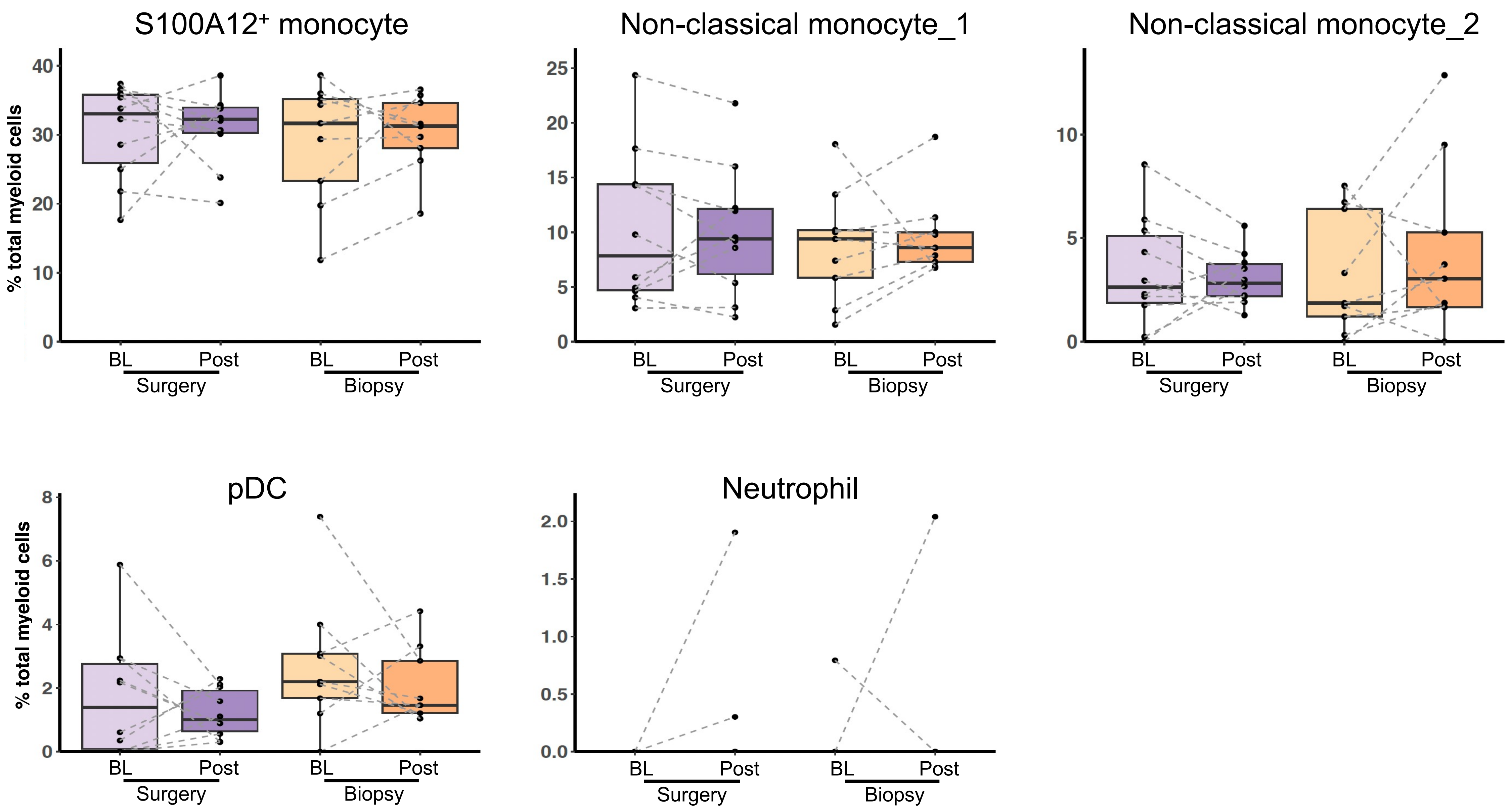
S100A12⁺ monocyte
Non-classical monocyte_1
Non-classical monocyte_2
pDC
Neutrophil
% total myeloid cells
% total myeloid cells
0
10
20
30
40
0
5
10
15
20
25
0
5
10
0
2
4
6
8
0.0
0.5
1.0
1.5
2.0
BL
Post
Surgery
BL
Post
Biopsy

Fig. S10. **Frequency of myeloid sub-clusters**.

Box plot of the frequency of other myeloid cell clusters from patients who underwent surgery (n = 10) or biopsy (n = 9) comparing post-surgery or post-biopsy samples to baseline (BL).

Table S1. Baseline patient characteristics by treatment arm

Characteristics, n (%)	Nivo (n=29)	Nivo+Bev (n=45)	Nivo+Ipi (n=30)
**Surgery**	14 (48)	16 (36)	13 (43)
**Age**			
Mean	58	63	62
Range	40-74	38-84	46-78
**Gender**			
Male	22 (76)	32 (71)	28 (93)
Female	7 (24)	13 (29)	2 (7)
**Race**			
White	21 (72)	38 (84)	26 (87)
Black	2 (7)	1 (2)	3 (10)
Asian	0	2 (4)	0
American Indian	0	1 (2)	0
Other	6 (21)	3 (7)	1 (3)
**KPS**			
100	15 (52)	20 (44)	20 (67)
90	0	1 (2)	0
80	14 (48)	22 (49)	10 (33)
60	0	2 (4)	0
**IMDC prognostic risk**			
Favorable	0	3 (7)	2 (7)
Intermediate	25 (86)	30 (67)	22 (73)
Poor	4 (14)	12 (27)	6 (20)
**Prior systemic regimens**			
0	29 (100)	38 (84)	29 (97)
1	0	5 (11)	1 (3)
≥2	0	2 (4)	0
**Nephrectomy prior to trial**			
Yes	9 (31)	14 (31)	11 (37)
No	20 (69)	31 (69)	19 (63)
**Number of Metastatic Sites**			
1	5 (17)	5 (11)	5 (17)
2	15 (52)	17 (38)	8 (27)
3	6 (21)	11 (24)	7 (23)
4	2 (7)	6 (13)	4 (13)
5	1 (3)	4 (9)	4 (13)
6	0 (0)	2 (4)	1 (3)
7	0 (0)	0 (0)	1 (3)
**Metastatic Sites**			
Lung	22 (76)	31 (69)	22 (73)
Liver	6 (21)	10 (22)	8 (27)
LN	19 (66)	31 (69)	19 (63)
Bone	7 (24)	17 (38)	14 (47)
Other	10 (34)	25 (56)	16 (53)
**Sarcomatoid**			
Yes	1 (3)	3 (7)	4 (13)

KPS: Karnofsky performance status; IMDC: International metastatic renal cell carcinoma database consortium

Table S2. Adverse events in any arm regardless of therapy attribution.

Adverse Event	Total (n=104)				Nivo (n=29)				Nivo+Bev (n=45)				Nivo+Ipi (n=30)			
	All		Grade 3+		All		Grade 3+		All		Grade 3+		All		Grade 3+	
	N	(%)	N	(%)	N	(%)	N	(%)	N	(%)	N	(%)	N	(%)	N	(%)
**Any Event**	**104**	**(100)**	**74**	**(71)**	**29**	**(100)**	**19**	**(66)**	**45**	**(100)**	**34**	**(76)**	**30**	**(100)**	**21**	**(70)**
Lipase increased	43	(41)	20	(19)	11	(38)	6	(21)	18	(40)	7	(16)	14	(47)	7	(23)
Hypertension	20	(19)	13	(13)	2	(7)	1	(3)	15	(33)	10	(22)	3	(10)	2	(7)
Pain	63	(61)	8	(8)	18	(62)	3	(10)	24	(53)	3	(7)	21	(70)	2	(7)
Fatigue	72	(69)	7	(7)	21	(72)	1	(3)	30	(67)	4	(9)	21	(70)	2	(7)
Lymphocyte decrease	37	(36)	7	(7)	13	(45)	1	(3)	12	(27)	3	(7)	12	(40)	3	(10)
Amylase increased	36	(35)	7	(7)	7	(24)	1	(3)	17	(38)	3	(7)	12	(40)	3	(10)
Hypercalcemia	31	(30)	6	(6)	12	(41)	1	(3)	11	(24)	2	(4)	8	(27)	3	(10)
Anemia	62	(60)	5	(5)	21	(72)	3	(10)	26	(58)	2	(4)	15	(50)	0	(0)
Hyperglycemia	62	(60)	5	(5)	19	(66)	1	(3)	25	(56)	1	(2)	18	(60)	3	(10)
ALT increased	43	(41)	4	(4)	13	(45)	1	(3)	17	(38)	2	(4)	13	(43)	1	(3)
Neoplasms malignant	7	(7)	5	(5)	3	(10)	1	(3)	4	(9)	4	(9)	0	(0)	0	(0)
AST increased	44	(42)	4	(4)	15	(52)	1	(3)	18	(40)	2	(4)	11	(37)	1	(3)
Dyspnea	43	(41)	4	(4)	9	(31)	0	(0)	21	(47)	3	(7)	13	(43)	1	(3)
Hyponatremia	32	(31)	4	(4)	10	(34)	2	(7)	9	(20)	1	(2)	13	(43)	1	(3)
Glycosuria	6	(6)	4	(4)	3	(10)	2	(7)	1	(2)	0	(0)	2	(7)	2	(7)
Proteinuria	51	(49)	3	(3)	20	(69)	2	(7)	22	(49)	1	(2)	9	(30)	0	(0)
Abdominal pain	35	(34)	3	(3)	10	(34)	1	(3)	20	(44)	2	(4)	5	(17)	0	(0)
Bilirubin increase	13	(13)	3	(3)	8	(28)	1	(3)	4	(9)	2	(4)	1	(3)	0	(0)
Nausea	43	(41)	2	(2)	11	(38)	0	(0)	22	(49)	1	(2)	10	(33)	1	(3)
Muscle weakness	18	(17)	2	(2)	4	(14)	0	(0)	8	(18)	1	(2)	6	(20)	1	(3)
Hypokalemia	12	(12)	2	(2)	3	(10)	0	(0)	7	(16)	1	(2)	2	(7)	1	(3)
Hypophosphatemia	12	(12)	2	(2)	6	(21)	2	(7)	2	(4)	0	(0)	4	(13)	0	(0)
Bone pain	8	(8)	2	(2)	2	(7)	0	(0)	5	(11)	1	(2)	1	(3)	1	(3)
Lung infection	6	(6)	2	(2)	0	(0)	0	(0)	6	(13)	2	(4)	0	(0)	0	(0)
Creatinine increased	61	(59)	1	(1)	16	(55)	0	(0)	27	(60)	1	(2)	18	(60)	0	(0)
Rash	51	(49)	1	(1)	13	(45)	0	(0)	19	(42)	1	(2)	19	(63)	0	(0)
Hyperkalemia	41	(39)	1	(1)	14	(48)	0	(0)	15	(33)	1	(2)	12	(40)	0	(0)
Diarrhea	40	(38)	1	(1)	14	(48)	0	(0)	18	(40)	1	(2)	8	(27)	0	(0)
Hyperuricemia	39	(38)	1	(1)	12	(41)	1	(3)	16	(36)	0	(0)	11	(37)	0	(0)
ALK Phos increased	36	(35)	1	(1)	11	(38)	1	(3)	15	(33)	0	(0)	10	(33)	0	(0)
Insomnia	33	(32)	1	(1)	9	(31)	1	(3)	13	(29)	0	(0)	11	(37)	0	(0)
Anorexia	29	(28)	1	(1)	7	(24)	0	(0)	15	(33)	1	(2)	7	(23)	0	(0)
Vomiting	27	(26)	1	(1)	9	(31)	0	(0)	12	(27)	0	(0)	6	(20)	1	(3)
Myalgia	19	(18)	1	(1)	7	(24)	0	(0)	7	(16)	1	(2)	5	(17)	0	(0)
Mucositis oral	10	(10)	1	(1)	3	(10)	0	(0)	6	(13)	1	(2)	1	(3)	0	(0)
Pleural effusion	10	(10)	1	(1)	2	(7)	1	(3)	6	(13)	0	(0)	2	(7)	0	(0)
Gait disturbance	9	(9)	1	(1)	3	(10)	1	(3)	4	(9)	0	(0)	2	(7)	0	(0)
Cough	55	(53)	0	(0)	15	(52)	0	(0)	23	(51)	0	(0)	17	(57)	0	(0)
Allergic rhinitis	41	(39)	0	(0)	10	(34)	0	(0)	18	(40)	0	(0)	13	(43)	0	(0)
Dizziness	38	(37)	0	(0)	11	(38)	0	(0)	16	(36)	0	(0)	11	(37)	0	(0)
Arthralgia	36	(35)	0	(0)	7	(24)	0	(0)	16	(36)	0	(0)	13	(43)	0	(0)
Edema	34	(33)	0	(0)	14	(48)	0	(0)	13	(29)	0	(0)	7	(23)	0	(0)
Nasal congestion	34	(33)	0	(0)	11	(38)	0	(0)	13	(29)	0	(0)	10	(33)	0	(0)
Constipation	33	(32)	0	(0)	12	(41)	0	(0)	14	(31)	0	(0)	7	(23)	0	(0)
Headache	33	(32)	0	(0)	8	(28)	0	(0)	15	(33)	0	(0)	10	(33)	0	(0)
Pruritus	31	(30)	0	(0)	11	(38)	0	(0)	10	(22)	0	(0)	10	(33)	0	(0)
Hypomagnesemia	30	(29)	0	(0)	9	(31)	0	(0)	15	(33)	0	(0)	6	(20)	0	(0)
Hematuria	29	(28)	0	(0)	10	(34)	0	(0)	13	(29)	0	(0)	6	(20)	0	(0)
Fever	28	(27)	0	(0)	10	(34)	0	(0)	13	(29)	0	(0)	5	(17)	0	(0)
Hypothyroidism	27	(26)	0	(0)	6	(21)	0	(0)	10	(22)	0	(0)	11	(37)	0	(0)
Hypoalbuminemia	25	(24)	0	(0)	8	(28)	0	(0)	13	(29)	0	(0)	4	(13)	0	(0)

Adverse Event	Total (n=104)				Nivo (n=29)				Nivo+Bev (n=45)				Nivo+Ipi (n=30)			
	All		Grade 3+		All		Grade 3+		All		Grade 3+		All		Grade 3+	
	N	(%)	N	(%)	N	(%)	N	(%)	N	(%)	N	(%)	N	(%)	N	(%)
TSH increased	23	(22)	0	(0)	9	(31)	0	(0)	10	(22)	0	(0)	4	(13)	0	(0)
Anxiety	22	(21)	0	(0)	7	(24)	0	(0)	11	(24)	0	(0)	4	(13)	0	(0)
Postnasal drip	20	(19)	0	(0)	9	(31)	0	(0)	7	(16)	0	(0)	4	(13)	0	(0)
Sore throat	20	(19)	0	(0)	5	(17)	0	(0)	10	(22)	0	(0)	5	(17)	0	(0)
Hypernatremia	18	(17)	0	(0)	8	(28)	0	(0)	5	(11)	0	(0)	5	(17)	0	(0)
Hoarseness	17	(16)	0	(0)	4	(14)	0	(0)	7	(16)	0	(0)	6	(20)	0	(0)
Tot. protein increased	16	(15)	0	(0)	5	(17)	0	(0)	8	(18)	0	(0)	3	(10)	0	(0)
Urinary frequency	16	(15)	0	(0)	6	(21)	0	(0)	6	(13)	0	(0)	4	(13)	0	(0)
Chills	15	(14)	0	(0)	5	(17)	0	(0)	6	(13)	0	(0)	4	(13)	0	(0)
Dysgeusia	15	(14)	0	(0)	4	(14)	0	(0)	9	(20)	0	(0)	2	(7)	0	(0)
LDH increased	14	(13)	0	(0)	2	(7)	0	(0)	8	(18)	0	(0)	4	(13)	0	(0)
Dry skin	13	(13)	0	(0)	6	(21)	0	(0)	6	(13)	0	(0)	1	(3)	0	(0)
WBC decreased	13	(13)	0	(0)	7	(24)	0	(0)	2	(4)	0	(0)	4	(13)	0	(0)
Blurred vision	12	(12)	0	(0)	3	(10)	0	(0)	4	(9)	0	(0)	5	(17)	0	(0)
Hyperthyroidism	12	(12)	0	(0)	1	(3)	0	(0)	5	(11)	0	(0)	6	(20)	0	(0)
Platelet decrease	11	(11)	0	(0)	3	(10)	0	(0)	6	(13)	0	(0)	2	(7)	0	(0)
Hypocalcemia	10	(10)	0	(0)	4	(14)	0	(0)	5	(11)	0	(0)	1	(3)	0	(0)
Weight loss	10	(10)	0	(0)	3	(10)	0	(0)	6	(13)	0	(0)	1	(3)	0	(0)
GERD	9	(9)	0	(0)	2	(7)	0	(0)	6	(13)	0	(0)	1	(3)	0	(0)
Hot flashes	9	(9)	0	(0)	3	(10)	0	(0)	6	(13)	0	(0)	0	(0)	0	(0)
Night sweats	9	(9)	0	(0)	4	(14)	0	(0)	3	(7)	0	(0)	2	(7)	0	(0)
Dry mouth	8	(8)	0	(0)	5	(17)	0	(0)	2	(4)	0	(0)	1	(3)	0	(0)
T3 decreased	8	(8)	0	(0)	3	(10)	0	(0)	3	(7)	0	(0)	2	(7)	0	(0)
BUN increased	7	(7)	0	(0)	3	(10)	0	(0)	0	(0)	0	(0)	4	(13)	0	(0)
Dry eye	7	(7)	0	(0)	3	(10)	0	(0)	2	(4)	0	(0)	2	(7)	0	(0)
Flu like symptoms	7	(7)	0	(0)	1	(3)	0	(0)	1	(2)	0	(0)	5	(17)	0	(0)
Hypoglycemia	7	(7)	0	(0)	1	(3)	0	(0)	5	(11)	0	(0)	1	(3)	0	(0)
Shoulder pain	7	(7)	0	(0)	3	(10)	0	(0)	1	(2)	0	(0)	3	(10)	0	(0)
Sneezing	7	(7)	0	(0)	2	(7)	0	(0)	5	(11)	0	(0)	0	(0)	0	(0)
T4 decreased	7	(7)	0	(0)	6	(21)	0	(0)	0	(0)	0	(0)	1	(3)	0	(0)
T4 increased	7	(7)	0	(0)	3	(10)	0	(0)	2	(4)	0	(0)	2	(7)	0	(0)
Hemoglobinuria	6	(6)	0	(0)	3	(10)	0	(0)	1	(2)	0	(0)	2	(7)	0	(0)
Multipoint pain	6	(6)	0	(0)	1	(3)	0	(0)	5	(11)	0	(0)	0	(0)	0	(0)
Phosphorus increased	6	(6)	0	(0)	3	(10)	0	(0)	2	(4)	0	(0)	1	(3)	0	(0)
Productive cough	6	(6)	0	(0)	3	(10)	0	(0)	1	(2)	0	(0)	2	(7)	0	(0)
Cortisol decreased	5	(5)	0	(0)	3	(10)	0	(0)	2	(4)	0	(0)	0	(0)	0	(0)
Nocturia	3	(3)	0	(0)	3	(10)	0	(0)	0	(0)	0	(0)	0	(0)	0	(0)
TSH decreased	3	(3)	0	(0)	3	(10)	0	(0)	0	(0)	0	(0)	0	(0)	0	(0)

Table S3. Ninety-day postoperative complications

Complication	Clavien Grade	Number of Complications, n (%) Total n=43
Diarrhea	II	1 (2)
Failure to thrive	II	1 (2)
DVT	II	1 (2)
Hematoma requiring drain placement	IIIb	1 (2)
Pancreatic leak requiring drain placement	IIIb	1 (2)
Sepsis/Death	V	1 (2)

Table S4. Clinical response among patients treated with ICT plus surgery or ICT without surgery

Response Measure	All Arms (n=104)	Nivo (n=29)	Nivo+Bev (n=45)	Nivo+Ipi (n=30)
	n (%)	n (%)	n (%)	n (%)
**Best Overall Response (BOR)* at 12 weeks**				
All Patients	50/104 (48)	17/29 (59)	20/45 (44)	13/30 (43)
ICT plus Surgery Patients	34/43 (79)	12/14 (86)	13/16 (81)	9/13 (69)
ICT without Surgery Patients	16/61 (26)	5/15 (33)	7/29 (24)	4/17 (24)
**BOR at 12 weeks excluding surgery effect ** **				
All Patients	35/104 (34)	13/29 (45)	16/45 (36)	9/30 (30)
ICT plus Surgery Patients	22/43 (51)	8/14 (57)	9/16 (56)	5/13 (38)

* BOR was defined as the response status consisting of either complete response (CR) or partial response (PR).
** Since some patients (N=8) had surgery removing target lesions before the 12-week assessment, BOR excluding surgery effect was based on assessment of the remaining target lesions. For those patients (N=4) with only one metastatic lesion resected by metastasectomy, BOR was based upon the 8-week response before surgery. Surgery resected target lesions in 4 patients in the nivo arm, 4 patients in the nivo+bev arm and 4 patients in the nivo+ipi arm. SD: stable disease.

Table S5: Patient Characteristics by Surgical Status

Baseline Patient Characteristics		Cytoreductive Surgery n (%)	Without Surgery n (%)
**All**		**43 (100)**	**61 (100%)**
Age - median (min, max)	N=104	63(40, 84)	59 (38, 79)
Gender			
	F	6 (14%)	16 (26%)
	M	37 (86%)	45 (74%)
Race			
	American Indian	0 (0%)	1 (2%)
	Asian	0 (0%)	2 (3%)
	Black	5 (12%)	1 (2%)
	Other	5 (12%)	5 (8%)
	White	33 (77%)	52 (85%)
Ethnicity			
	Hispanic	5 (13%)	6 (12%)
	Not Hispanic	34 (87%)	46 (88%)
ECOG Status			
	0	25 (58%)	29 (48%)
	1	18 (42%)	30 (49%)
	2	0 (0%)	2 (3%)
KPS			
	60	0 (0%)	2 (3%)
	80	17 (40%)	29 (48%)
	90	0 (0%)	1 (2%)
	100	26 (60%)	29 (48%)
IMDC Risk Factor Score			
	0	1 (2%)	4 (7%)
	1	6 (14%)	15 (25%)
	2	27 (63%)	29 (48%)
	3	9 (21%)	7 (11%)
	4	0 (0%)	5 (8%)
	5	0 (0%)	1 (2%)
IMDC Risk Factor			
	Favorable	1 (2%)	4 (7%)
	Intermediate	33 (77%)	44 (72%)
	Poor	9 (21%)	13 (21%)
Prior Nephrectomy			
	Y	5 (12%)	29 (48%)
Prior Nephrectomy Side			
	Both	0 (0%)	1 (3%)
	Left	3 (60%)	12 (41%)
	Right	2 (40%)	16 (55%)
Prior Treatment			
	XRT	3 (7%)	8 (13%)
	Embolization	0 (0%)	2 (3%)
	Cryoablation	0 (0%)	1 (2%)

Baseline Patient Characteristics		Cytoreductive Surgery n (%)	Without Surgery n (%)
	Surgery	0 (0%)	2 (3%)
Number of Prior Systemic Therapies			
	0	42 (98%)	54 (89%)
	1	1 (2%)	5 (8%)
	2	0 (0%)	1 (2%)
	4	0 (0%)	1 (2%)
Prior Therapy			
	Sunitinib	0 (0%)	5 (8%)
	Pazopanib	1 (2%)	4 (7%)
	Axitinib	0 (0%)	1 (2%)
	Everolimus	0 (0%)	1 (2%)
Number of Sites			
	1	8 (19%)	7 (11%)
	2	21 (49%)	19 (31%)
	3	10 (23%)	14 (23%)
	4	4 (9%)	8 (13%)
	5	0 (0%)	9 (15%)
	6	0 (0%)	3 (5%)
	7	0 (0%)	1 (2%)
Metastasis Site			
	Lung	28 (65%)	47 (77%)
	Liver	7 (16%)	17 (28%)
	LN	26 (60%)	43 (70%)
	Bone	15 (35%)	24 (39%)
	Other	17 (40%)	34 (56%)
Sarcomatoid			

Table S6. Clinical trials evaluating surgery outcomes.

Era	Study	Median Follow-up (months)	Agents	n	Surgery (n)	Median OS (months)	HR OS (95% CI)
Checkpoint inhibitor	Present study (not randomized to surgery)	57.9	ICT plus Surgery	43	43	54.7 (2-year OS 84%)	N/A
			ICT without Surgery	61	0	23.5 (2-year OS 49%)	
			ICT plus Biopsy after prior Nephrectomy	17	0	54..6 (2-year OS 82%)	
			ICT plus Biopsy without prior Nephrectomy	19	0	24.4 (2-year OS 53%)	
			ICT Alone	25	0	9.1 (2-year OS 24%)	
Tyrosine Kinase Inhibitor	Méjean et al†	50.9	Surgery+Sunitinib		210	13.9	0.89 (0.71-1.1); p NR
			Sunitinib		38	18.4	
	Bex et al†	39.6	Surgery+Sunitinib		46	15	0.57 (0.34-0.95); p=0.03
			Sunitinib+Delayed Surgery		34	32.4	
Cytokine	Flanigan et al†	NR	Surgery+IFN alfa		141	13.6	0.69 (0.55-0.87); p=0.002
			IFN alfa		0	7.8	
	Flanigan et al†	12.1	Surgery+IFN alfa		113	11.1	p=0.05‡
			IFN alfa		0	8.1	
	Mickisch et al†	NR	Surgery+IFN alfa		38	17	p=0.03‡
			IFN alfa		0	7	

OS: overall survival; HR: hazard ratio; CI: confidence interval; ICT: immune checkpoint therapy; IFN: interferon
† Outcomes reported according to the intention-to-treat population.
‡ Hazard ratio not reported. P-value derived from stratified log rank test.

Table S7. IFN-γ signature genes and TLS signature genes

**IFN-γ 25 gene signature**	**TLS 24 gene signature**
CCL5	CCL3
CD8A	CCL4
GZMA	CCL5
LAG3	CCL19
GZMB	CCL21
PRF1	CXCL9
CD27	CXCL10
CXCL13	CXCL11
CCR5	CXCL13
CD2	CD19
IL2RB	CD79A
IL2RG	MS4A1
CD3D	CXCR5
CXCR6	CCR7
CD4	LAMP3
CD74	CD86
IDO1	CD4
STAT1	CD3D
CXCL10	PTPRC
CXCL9	FCER2
PTPRC	PDCD1
PDCD1	CD40
TBX21	CD8A
CXCL11	CD79B
IFNG	

Table S8. Antibodies panel for CODEX Assay

Antibody	Clone	Brand	Cat#	Barcode	Reporter & Fluorochrome	Dilution	Incubation Mode	Exposure (ms)
CD107A	H4A3	Akoya	4350001	BX006	RX006-Cy5	1:200	O/N	500
CD11C	118/A5	Akoya	4350020	BX024	RX024-Cy5	1:100	3 hrs.	500
CD15	HI98	BioLegend	301902	BX033	RX033-Cy5	1:2000	O/N	500
CD20	L26	Akoya	4150018	BX007	RX007-AF488	1:100	3 hrs.	250
CD21	EP3093	Akoya	4450027	BX032	RX032-ATTO550	1:200	3 hrs.	333
CD23	EPR3617	Abcam	ab247568	BX041	RX041-ATTO550	1:100	O/N	333
CD3	UCHT1	Dako	A0452	BX020	RX020-ATTO550	1:100	3 hrs.	333
CD31	EP3095	Akoya	4150017	BX001	RX001-AF488	1:200	O/N	250
CD4	EPR6855	Abcam	ab181724	BX010	RX010-AF488	1:50	O/N	300
CD44	IM7	Akoya	4250002	BX005	RX005-ATTO550	1:200	O/N	333
CD45RO	UCHL1	Akoya	4250023	BX017	RX017-ATTO550	1:200	O/N	500
CD47	Polyclonal	R&D	AF4670	BX042	RX042-Cy5	1:200	O/N	500
CD68	KP1	Akoya	4350019	BX015	RX015-Cy5	1:200	O/N	500
CD8	C8/144B	Akoya	4250012	BX026	RX026-ATTO550	1:800	O/N	333
E-CADHERIN	4A2C7	Akoya	4250021	BX014	RX014-ATTO550	1:200	O/N	333
EOMES	WD1928	Thermofisher	14-4877-82	BX035	RX035-ATTO550	1:50	O/N	500
FOXP3	PCH101	Thermofisher	14-4776-82	BX031	RX031-AF488	1:50	3 hrs.	300
HLA-DR	EPR3692	Abcam	ab215985	BX037	RX037-AF488	1:100	O/N	250
ICOS	D1K2T	CST-Custom	89601BF	BX030	RX030-Cy5	1:20	O/N	500
KI67	B56	Akoya	4250019	BX047	RX047-ATTO550	1:500	O/N	333
LAG3	17B4	Lifespan	C18692	BX036	RX036-Cy5	1:100	O/N	500
MMP9	L51/82	BioLegend	819701	BX027	RX027-Cy5	1:50	O/N	500
PAN-CYTOKERATIN	AE-1/AE-3	Akoya	4150020	BX019	RX019-AF488	1:200	O/N	500
PD-1	D4W2J	CST-Custom	86163BF	BX016	RX016-AF488	1:25	O/N	300
T-BET	4B10	BioLegend	644802	BX043	RX043-AF488	1:25	3 hrs.	250

Supplementary Files

This is a list of supplementary files associated with this preprint. Click to download.

- nrreportingsummaryPS.pdf
- TABLES8DEGs.xlsx
